# Novel similarity measures under complex pythagorean fuzzy soft matrices and their application in decision making problems

**DOI:** 10.1038/s41598-024-65324-6

**Published:** 2024-07-25

**Authors:** Muhammad Zeeshan, Madad Khan, Ramsha Shafqat, Ali Althobaiti, Saad Althobaiti, Tola Bekene Bedada

**Affiliations:** 1 Department of Mathematics, The University of Agriculture, Dera Ismail Khan, Pakistan; 2 Department of Mathematics, COMSATS University Islamabad, Abbottabad Campus, Abbottabad, Pakistan; 3 Department of Mathematics and Statistics, The University of Lahore, Sargodha, 40100 Pakistan; 4 Department of Mathematics, College of Science, Taif University, P.O. Box 11099, 21944 Taif, Saudi Arabia; 5 Department of Sciences and Technology, Ranyah University Collage, Taif University, P.O. Box 11099, 21944 Taif, Saudi Arabia; 6 Department of Mathematics, Wachemo University, Hosaina , Ethiopia

**Keywords:** Soft set, Complex Pythagorean soft set, Complex Pythagorean fuzzy soft matrices, Decision-making, Engineering, Mathematics and computing

## Abstract

Complex fuzzy soft matrices play a crucial role in various applications, including decision-making, pattern recognition, signals processing, and image processing. The main objective of this study is to introduce the unique notions of complex Pythagorean fuzzy soft matrices (CPFSMs), which provide more flexibility and accuracy in modelling uncertainty. CPFSMs incorporate Pythagorean fuzzy soft matrices, allowing for more sophisticated uncertainty modeling. The key findings of CPFSMs, specific instances, and certain fundamental set-theoretic operations and principles were covered. A set of new distance metrics between two CPFSMs has been defined. In the context of complex Pythagorean fuzzy soft sets and complex Pythagorean fuzzy soft matrices, we created a CPFS decision-making technique. Moreover, the application’s numerical example and comparison analysis have been effectively demonstrated. Thus, by integrating the concepts of Pythagorean fuzzy sets, soft matrices, and complex numbers, CPFSMs provide a robust framework with membership and non-membership degrees for complex decision-making modeling and analyzing uncertain data.

## Introduction

Many theories in the literature are used to deal with the ambiguity and uncertainty of many problems that arise in engineering, social science, economics, and other fields of science. However, because of the parametrization tool used, all of the theories have their intuitive limitations. Molodstov^1^ discussed a new mathematical framework, known as soft set (SS), devoid of parametrization inadequacies, tailored for handling uncertainties, and discussed several findings stemming from its application. Maji et al.^2^ studied soft set theory and determined some binary operations on SSs such as intersection, union, complement, OR, AND, and equality of SSs. Rehman et al.^3^ Discussed fuzzy soft sets (FSSs) and FS lattices. Using fuzzy sets (FSs) and FSSs, soft hypermodules and fuzzy soft hypermodules were proposed by Ameri^4^. Rehman et al.^5^ defined some new type of graphs called neighborhood-based soft covering rough graphs.

Maji et al.^6,7^ proceeded on to successfully extend the concept of SS to FSS and intuitionistic FSS, as well as discuss their applications in decision-making (DM) problems. Zeb et al.^8^ discussed applications of fermatean fuzzy soft aggregation operators in symptomatic treatment of COVID-19 (a case study of patient identification).

The concept of Pythagorean fuzzy soft set (PFSS) was proposed by Peng et al.^9^. They reviewed different operations on it and proposed a DM algorithm based on PFSSs. FSSs and intuitionistic FSSs are special cases of a PFSS, as shown by their structure. Some novel distance measures for the PFSSs were introduced by Athira et al.^10^. Moreover, to compute the degree of fuzziness of the set, Athira et al.^11^ discussed some new entropy measures in the environment of PFSs. They discussed their applications in DM problems. Naeem et al.^12^ presented an algorithm and flow chart based on PFSSs for multi-criteria group decision-making (MCGDM) in the context of site selection. As extensions to the TOPSIS and VIKOR, they established the PFS TOPSIS and the PFS VIKOR methods. They discussed an application addressing the stock exchange investment challenge and tackled it using PFS TOPSIS and PFS VIKOR methodologies. Zulqarnain et al.^13^ discussed the desirable axioms of PFS weighted averaging and PFS weighted geometric operators. They developed DM algorithms based on the proposed operators. In Zhang et al.^14^ gave the idea of a PF N-soft set, a generalization of the N-soft set. They discussed related operations to this theory, such as extended intersection and union, weak complement, and restricted intersection and union. Moreover, two algorithms for dealing with MCGDM problems were introduced in the environment of PF N-soft sets. Jia-hua gave the theory of possibility PFSSs in^15^. They identified various binary operations on the possibility of PFSSs. They introduced a similarity measure to compare two possible PFSSs and presented their applications in DM problems. Zeb et al.^16^ introduced aggregation operators of Pythagorean fuzzy bi-polar soft sets and discussed their applications in multiple attribute decision-making problems.

N. Çağman and S. Enginoğlu have applied soft matrices and explored their various properties. They proposed a soft max-min DM algorithm, introducing a valuable tool for addressing uncertainties^17^. Moreover, fuzzy soft matrices (FSMs) have been defined alongside the fs-max-min DM method, discussing successful application in DM scenarios fraught with ambiguity^18^. Indeed, they provided a new direction for both practical applications and foundational research within the realm of SSs and FSSs theory. Naim and Serdar^19^ discussed soft matrices, which serve as representations of Molodtsov’s SSs, and applied them to resolve DM challenges. Khan et al.^20^ defined complex fuzzy soft matrices and discussed some new operations on these matrices. Furthermore, they constructed an algorithm utilizing complex fuzzy soft matrices and applied it in a signal-processing DM scenario. Yong and Chenli^21^ generalized the matrix representation of SSs to FSSs, while Chetia and Das^22^ expanded it to intuitionistic fuzzy soft matrices. They subsequently utilized the proposed extensions to address DM problems. Guleria et al.^23^ presented the notion of PFS matrices and studied several properties and binary operations associated with these matrices. Furthermore, they discussed the applications of PFS matrices in DM scenarios and medical diagnosis. Akram et al.^24^ discussed a new decision-making methodology, namely, the complex Pythagorean fuzzy method. They introduced some novel operators in the environment of complex Pythagorean fuzzy N-soft sets and utilized them in MCGDM problems. Zeb et al.^25^ defined some generalized approach based on Hamacher aggregation operators for q-rung orthopair fuzzy soft sets. They discussed their applications in optimizing decision-making in electric power system selection.

The main objective of this paper is to introduce the novel concepts of CPFSMs. CPFSMs offer a powerful and flexible mathematical framework for modeling and solving complex decision-making problems, enabling the effective handling of uncertainty, ambiguity, and multi-criteria decision-making challenges. By integrating Pythagorean fuzzy sets, soft matrices, and complex numbers, CPFSMs provide a robust and efficient tool for decision-makers to analyze and optimize uncertain and complex systems, leading to more informed and accurate decision-making outcomes in various fields, including management, economics, engineering, and healthcare.

### Motivation

CPFSMs are a specialized extension of soft matrices, fuzzy soft matrices, complex fuzzy soft matrices, and Pythagorean fuzzy soft matrices designed to handle ambiguity and uncertainty in DM processes. They combine elements of soft sets, fuzzy sets, and complex numbers to establish a new approach for representing and analyzing uncertain, vague, and complex information. The motivation behind using CPFSMs lies in their ability to model and handle complex systems where traditional soft matrices fail to discuss the intricacies of uncertainty. Here are some significant motivations for utilizing CPFSMs:

1. Real-world challenges frequently involve uncertainty and ambiguity. CPFSMs provide a flexible representation that can better accept uncertainty in DM procedures than the traditional soft matrices.
2. Decision-making procedures frequently require integrating data from various sources, each with its level of uncertainty. CPFSMs allow for integrating multiple sources of information by offering a uniform representation framework.
3. CPFSMs are based on rigorous mathematical foundations, leveraging concepts from FS theory, SS theory, and complex numbers. This mathematical framework ensures the reliability and consistency of the analysis conducted by applying these matrices.
4. CPFSMs find applications in diverse domains such as DM, pattern recognition, image processing, expert systems, and artificial intelligence. Their ability to discuss uncertain and complex information makes them useful tools in addressing real-life problems across various fields.

### Contributions

CPFSMs have emerged as a powerful tool for addressing decision-making problems in complex and uncertain environments. CPFSMs enables decision-makers to effectively handle ambiguity and uncertainty by integrating Pythagorean fuzzy sets, soft sets, and complex numbers, allowing for the simultaneous consideration of multiple criteria and uncertainty levels. This approach facilitates more accurate and informed decision-making outcomes, particularly in complex and dynamic environments.

The paper is organized as follows. A formal definition of the CPFS set is provided in "Preliminaries". In "Complex pythagorean fuzzy soft matrix theory" section, we introduce a new concept of a CPFSM. Some particular examples and basic definitions of CPFSMs are also presented in this section. In "Main results" section, we discussed the main results of CPFSMs. "Distance measures of complex Pythagorean fuzzy soft matrices" section is concerned with the distance measures of CPFSMs. In section 5, we developed the DM method. The comparison analysis of the proposed CPFSMs is discussed in "Applications of complex Pythagorean fuzzy soft matrix in decision-making" section. In "Comparison analysis" and "Conclusion"section, we discussed the advantages and conclusion, respectively.

## Preliminaries

We will discuss here the basic set-theoretic operations and laws of CPFS sets and matrices and also discuss particular examples of these operations and laws.

### Definition 2.1

^1^ Let *X* be an initial universe set and $\Gamma$ be a set of parameters. Let *P*(*X*) denote the power set of *X*. Consider a nonempty set $\tau ,$ $\tau \subset \Gamma$. A pair$(\digamma ,\Gamma )$ is called a soft set over *X*, where $\digamma$ is a mapping given by $\digamma :\tau \rightarrow P(X)$.

### Definition 2.2

^15^ Let $\digamma (X)$ denotes the set of all fuzzy sets of *X*. A pair $(\digamma ,\Gamma )$ is called a fuzzy soft set over $\digamma (X)$, where $\digamma$ is a mapping given by $\digamma :\tau \rightarrow P(\digamma (X)).$

### Definition 2.3

^15^ The pair $(\digamma ,\Gamma )$ is called the Pythagorean fuzzy soft set (PFSS) over *X* if $\digamma  :\tau  \to PFS(X)$ and can be represented as

$$(\digamma ,\Gamma )=\{(q,\digamma (q)):q\in \tau \digamma (q)\in PFS(X)\},$$

where *PFS*(*X*) denotes the set of all Pythagorean fuzzy sets of *X*.

### Definition 2.4

^26^ Let $X=\{ \tilde{a}_{1},\tilde{a}_{2},...,\tilde{a}_{n} \}$ be the universal set and $\Gamma =\{q_{1},q_{2},...,q_{n} \}$ be the set of parameters. If $\tau \subset \Gamma ,$ then $(\psi ,\tau )$ is a CPFS set over *X*,  where $\psi$ is a mapping given by $\psi :\tau \rightarrow P(\lambda _{U}),$ and is defined as

$$\begin{aligned} \psi (\tilde{a}_{t})=\{q_{i},\varkappa (\tilde{a}_{t})e^{i\epsilon (\tilde{a}_{t})},\ell (\tilde{a}_{t})e^{i\kappa (\tilde{a}_{t})}:\tilde{a}_{t}\in X,q_{i}\in \tau \}, \end{aligned}$$

### Remark 2.1

For a given set if $\varkappa _{j}(\tilde{a}_{i})=0=\kappa _{j}(\tilde{a}_{i})$ The CPFS set is then reduced to the Pythagorean fuzzy soft set.

### Definition 2.5

^26^ Let $(\digamma ,\tau )$ and $(\digamma ^{\prime },\tau ^{\prime })$ be two CPFS sets over *X*, where $\tau ,\tau ^{\prime }\subset \Gamma .$ Then

(*i*).: $(\digamma ,\tau )\subseteq (\digamma ^{\prime },\tau ^{\prime } )$ if $\tau \subseteq \tau ^{\prime }$ and $\varkappa _{\digamma (q_{i})}(\tilde{a}_{i})\le \varkappa _{\digamma ^{\prime }(q_{i} )}(\tilde{a}_{i}),\ell _{\digamma (q_{i})}(\tilde{a}_{i})\ge \ell _{\digamma ^{\prime }(q_{i})}(\tilde{a}_{i})$ for amplitude terms and $\epsilon _{\digamma (q_{i})}(\tilde{a}_{i})\le \epsilon _{\digamma ^{\prime }(q_{i})}(\tilde{a}_{i}),\kappa _{\digamma (q_{i})}(\tilde{a} _{i})\ge \kappa _{\digamma ^{\prime }(q_{i})}(\tilde{a}_{i})$ for phase terms, for all $\tilde{a}_{t}\in X.$
(*ii*).: $(\digamma ,\tau )=(\digamma ^{\prime },\tau ^{\prime })$ if and if $\varkappa _{\digamma (q_{i})}(\tilde{a}_{i})=\varkappa _{\digamma ^{\prime }(q_{i})}(\tilde{a}_{i}),\ell _{\digamma (q_{i} )}(\tilde{a}_{i})=\ell _{\digamma ^{\prime }(q_{i})}(\tilde{a}_{i})$ for amplitude terms and $\epsilon _{\digamma (q_{i})}(\tilde{a}_{i})=\epsilon _{\digamma ^{\prime }(q_{i})}(\tilde{a}_{i}),\kappa _{\digamma (q_{i})}(\tilde{a}_{i})=\kappa _{\digamma ^{\prime }(q_{i})}(\tilde{a}_{i} )$ for phase terms.
(*iii*).: $(\digamma ^{c},\tau )$ is the complement of $(\digamma ,\tau )$ where

$$\begin{aligned} \digamma ^{c}=\{(q_{i},\ell (\tilde{a}_{t})e^{i\kappa (\tilde{a}_{t})},\varkappa (\tilde{a}_{t})e^{i\epsilon (\tilde{a}_{t})}):\tilde{a}_{t}\in X,q_{i}\in \tau \}. \end{aligned}$$

## Complex Pythagorean fuzzy soft matrix theory

In this section, we introduce a novel concept: the introduction of a CPFSM. It’s notable to mention that a CPFSM surpasses a complex fuzzy soft matrix in generality. This is because each entry of the matrix incorporates both the degree of membership function and non-membership function, offering a more comprehensive selection in decision-making problems.

### Complex Pythagorean fuzzy soft matrix

Let $X=\{ \tilde{a}_{1},\tilde{a}_{2},...,\tilde{a}_{n}\}$ be the universal set and $\Gamma =\{q_{1},q_{2},...,q_{n}\}$ be the set of parameters. If $\tau \subset \Gamma ,$ then $(\psi ,\tau )$ is a CPFS set over *X*,  where $\psi$ is a mapping given by $\psi :\tau \rightarrow P(\lambda _{X}),$ and is defined as

$$\begin{aligned} \psi (\tilde{a}_{t})=\{q_{i},\varkappa (\tilde{a}_{t})e^{i\epsilon (\tilde{a}_{t})},\ell (\tilde{a}_{t})e^{i\kappa (\tilde{a}_{t})}:\tilde{a}_{t}\in X,q_{i}\in \tau \}, \end{aligned}$$

where $P(\lambda _{U})$ denotes the power sets of CPFSSs. Then, the CPFS sets can be expressed in matrix form as $[ {\mathcal {L}} ]_{\hat{s}\times \breve{u}}=[\mid a_{ij}\mid ]_{\hat{s}\times \breve{u}}$ for $i=1,2,...,\hat{s}$ and $j=1,2,...,\breve{u}$; where

1 $$ {\mid }a_{{ij}} {\mid } = \left\{ {\begin{array}{*{20}l}    {\xi _{{ij}} (\tilde{a}_{t} ) = \left( {q_{i} ,|\partial _{{ij}} (\tilde{a}_{t} )e^{{i\epsilon _{{ij}} (\tilde{a}_{t} )}} |,|\lambda |_{{ij}} (\tilde{a}_{t} )e^{{i\kappa _{{ij}} (\tilde{a}_{t} )}} |} \right)} \hfill & {if\,\,q_{i}  \in \tau } \hfill  \\    0 \hfill & {otherwise} \hfill  \\   \end{array} } \right..$$

Note that

$$\begin{aligned} \xi _{ij}(\tilde{a}_{t})&=[|\partial _{ij}(\tilde{a}_{t})e^{i\epsilon _{ij}(\tilde{a}_{t})}|,|\lambda |_{ij}(\tilde{a}_{t})e^{i\kappa _{ij} (\tilde{a}_{t})}|]\\&=[\varkappa _{ij}(\tilde{a}_{t}),\ell _{ij}(\tilde{a}_{t})], \end{aligned}$$

represents the elements of $[ {\mathcal {L}} ]_{\hat{s}\times \breve{u}}$ corresponding to the elements $\tilde{a}_{k}$ of *X* for $i=1,2,...,\hat{s}$ and $k,j=1,2,...,\breve{u}$ where $\varkappa _{ij}(\tilde{a}_{t}),\ell _{ij}(\tilde{a}_{t})\in [0,1]$.

Also, note that the elements $\varkappa _{ij}(\tilde{a}_{t})$ and $\ell _{ij}(\tilde{a}_{t})$ are known as truth grade (TG) and false grade (FG) of the CPFSM and $0\le (\varkappa _{ij}(\tilde{a}_{t}))^{2}+(\ell _{ij}(\tilde{a}_{t}))^{2}\le 1.$

Equation (1) can be rearranged as:

$$\begin{aligned} \begin{array}{c|cccccc} \xi _{ij}(\tilde{a}_{t}) &{} \tilde{a}_{1} &{} \tilde{a}_{2} &{}. &{}. &{}. &{} \tilde{a}_{\breve{u}}\\ \hline q_{1} &{} \xi _{11}(\tilde{a}_{1}) &{} \xi _{12}(\tilde{a}_{2}) &{}. &{}. &{}. &{} \xi _{1\breve{u}}(\tilde{a}_{\breve{u}})\\ q_{2} &{} \xi _{21}(\tilde{a}_{1}) &{} \xi _{22}(\tilde{a}_{2}) &{}. &{}. &{}. &{} \xi _{2\breve{u}}(\tilde{a}_{\breve{u}})\\ . &{}. &{}. &{}. &{}. &{}. &{}.\\ . &{}. &{}. &{}. &{}. &{}. &{}.\\ . &{}. &{}. &{}. &{}. &{}. &{}.\\ q_{\hat{s}} &{} \xi _{\hat{s}1}(\tilde{a}_{1}) &{} \xi _{\hat{s}2}(\tilde{a}_{2}) &{}. &{}. &{}. &{} \xi _{\hat{s}\breve{u}}(\tilde{a}_{\breve{u}}) \end{array} \ . \end{aligned}$$

We would represent this CPFS set in matrix form as:

$$\begin{aligned}{}[{\mathcal {L}} ]_{\hat{s}\times \breve{u}}= \begin{pmatrix} \xi _{11}(\tilde{a}_{1}) &{} \xi _{12}(\tilde{a}_{1}) &{}. &{}. &{}. &{} \xi _{1\breve{u}}(\tilde{a}_{\breve{u}})\\ \xi _{21}(\tilde{a}_{1}) &{} \xi _{22}(\tilde{a}_{2}) &{}. &{}. &{}. &{} \xi _{2\breve{u}}(\tilde{a}_{\breve{u}})\\ . &{}. &{}. &{}. &{}. &{}.\\ . &{}. &{}. &{}. &{}. &{}.\\ . &{}. &{}. &{}. &{}. &{}.\\ \xi _{\hat{s}1}(\tilde{a}_{1}) &{} \xi _{\hat{s}2}(\tilde{a}_{2}) &{}. &{}. &{}. &{} \xi _{\hat{s}\breve{u}}(\tilde{a}_{\breve{u}}) \end{pmatrix} \end{aligned}$$

where $$\xi _{ij}(\tilde{a}_{t})=[\varkappa _{ij}(\tilde{a}_{t}),\ell _{ij} (\tilde{a}_{t})].$$

An example of the newly defined CPFSM is given below.

### Why do we need this new model?

Although existing fuzzy soft matrices are highly effective in dealing with ambiguous information, their limitations highlight the need for a superior model that can serve as a powerful tool in dealing with ambiguous information. The principle of fuzzy soft matrices has been utilized in separated areas, but the principle of fuzzy soft matrices has limited applications due to its structure. Because if a person faces information in the form of TG and falsity grade (FG), then the fuzzy soft matrices principle has failed in certain actual life troubles. To overcome this difficulty, we introduce the concepts of CPFSMs. False grades in CPFSMs play a vital role in defining the features of CPFSMs. This term distinguishes a CPFSM from all other soft matrices in the literature. The CPFSMs discuss two-dimensional phenomena in the form of truth and false grades, making them superior to handling ambiguous and intuitive information prevalent in time-periodic phenomena. They provide a powerful and flexible framework for modeling and analyzing complex decision-making problems, which are increasingly prevalent in various fields, including management, economics, engineering, and healthcare. Traditional approaches often struggle to handle uncertainty, ambiguity, and multi-criteria decision-making challenges, leading to inaccurate or suboptimal decisions. CPFSMs address these limitations by integrating Pythagorean fuzzy sets, soft matrices, and complex numbers, enabling the simultaneous consideration of multiple criteria, uncertainty levels, and complex relationships. This allows for more accurate and informed decision-making outcomes, making CPFSMs a crucial tool for tackling complex real-world problems and enabling better decision-making in uncertain and dynamic environments.

#### Example 3.1

Assume that there are three iPhones under consideration, namely the universes $X=\{ \tilde{a}_{1}=iPhone$ $11,\tilde{a}_{2}=iPhone$ $12,\tilde{a} _{3}=iPhone$ 12 $pro\}$. Let $\Gamma =\{q_{1},q_{2},q_{3}\}$ be the set of parameters where $q_{1}$ stands for “beautiful”, $q_{2}$ stands for “cheap”, $q_{3}$ stands for “expensive”. Consider the mapping $\psi$ form parameter set $\tau =\{q_{1},q_{3}\} \subset \Gamma$ to the power set of all CPFS sets over *X*. Then,

$$\begin{aligned}{} & {} [\psi (\tilde{a}_{1})]=\{(q_{1},(0.2e^{i\frac{\pi }{2}},0.5e^{i\pi } )),(q_{3},(|0.2e^{i\frac{\pi }{2}}|,|0.5e^{i\pi }|))\}\\{} & {} [\psi (\tilde{a}_{2})]=\{(q_{1},(0.1e^{i\frac{3\pi }{2}},0.8e^{i2\pi } )),(q_{3},(|0.1e^{i\frac{3\pi }{2}}|,|0.8e^{i2\pi }|)\}\\{} & {} [\psi (\tilde{a}_{3})]=\{(q_{1},(0.6e^{i\frac{\pi }{2}},0.8e^{i\pi } )),(q_{3},0.8e^{i\frac{\pi }{4}}0.3e^{i\frac{\pi }{2}})\}. \end{aligned}$$

Now,

$$\begin{aligned}{} & {} [|\psi (\tilde{a}_{1})|]=\{(q_{1},(|0.2e^{i\frac{\pi }{2}}|,|0.5e^{i\pi }|)),(q_{3},(|0.2e^{i\frac{\pi }{2}}|,|0.5e^{i\pi }|))\}\\{} & {} [|\psi (\tilde{a}_{2})|]=\{(q_{1},(|0.1e^{i\frac{3\pi }{2}}|,|0.8e^{i2\pi }|)),(q_{3},(|0.1e^{i\frac{3\pi }{2}}|,|0.8e^{i2\pi }|)\}\\{} & {} [|\psi (\tilde{a}_{3})|]=\{(q_{1},(|0.6e^{i\frac{\pi }{2}}|,|0.8e^{i\pi }|)),(q_{3},|0.8e^{i\frac{\pi }{4}}|,|0.3e^{i\frac{\pi }{2}}|)\}, \end{aligned}$$

where

$$\begin{aligned} |0.2e^{i\frac{\pi }{2}}|= & {} |0.2(\cos \frac{\pi }{2}+i\sin \frac{\pi }{2})|\\= & {} |0.2(0+i)|=|0.2i|\\= & {} 0.2\\ |0.5e^{i\pi }|= & {} |0.5(\cos \pi +i\sin \pi )|=|0.5(-1+0.i)|=|-0.5|\\= & {} 0.5\\ |0.1e^{i\frac{3\pi }{2}}|= & {} |0.1(\cos \frac{3\pi }{2}+i\sin \frac{3\pi }{2})|\\= & {} |0.1(0+i(-1))|\\= & {} |-0.1i|\\= & {} 0.1\\ |0.8e^{i2\pi }|= & {} |0.8(\cos 2\pi +i\sin 2\pi )|=|0.8(1+0.i)|\\= & {} |0.8|\\= & {} 0.8\\ |0.6e^{i\frac{\pi }{2}}|= & {} |0.6(\cos \frac{\pi }{2}+i\sin \frac{\pi }{2})|\\= & {} |0.6(0+i)| \\= & {} |0.6i|\\= & {} 0.6\\ |0.8e^{i\pi }|= & {} |0.8(\cos \pi +i\sin \pi )|=|0.8(-1+0.i)|=|-0.8|\\= & {} 0.8\\ |0.5e^{i\frac{3\pi }{2}}|= & {} |0.5(\cos \frac{3\pi }{2}+i\sin \frac{3\pi }{2})| \\= & {} |0.5(0-i)|\\= & {} |-0.5i|\\= & {} 0.5\\ |0.7e^{i\pi }|= & {} |0.7(\cos \pi +i\sin \pi )|=|0.7(-1+0.i)|\\= & {} |-0.7|\\= & {} 0.7\\ |0.4e^{i2\pi }|= & {} |0.4(\cos 2\pi +i\sin 2\pi )|=|0.4(1+0.i)|\\= & {} |0.4|\\= & {} 0.4\\ |0.6e^{i\frac{\pi }{2}}|= & {} |0.6(\cos \frac{\pi }{2}+i\sin \frac{\pi }{2})|\\= & {} |0.6(0+1.i)|\\= & {} |0.6i|\\= & {} 0.6\\ |0.8e^{i\frac{\pi }{4}}|= & {} |0.8(\cos \frac{\pi }{4}+i\sin \frac{\pi }{4})|\\= & {} |0.8(\frac{1}{\sqrt{2}}+i\frac{1}{\sqrt{2}})|\\= & {} |0.8(0.707+i0.707)|\\= & {} |0.75+i0.57|\\= & {} \sqrt{(0.57)^{2}+(0.57)^{2}}\\= & {} 0.8\\ |0.3e^{i\frac{\pi }{2}}|= & {} |0.3(\cos \frac{\pi }{2}+i\sin \frac{\pi }{2})|\\= & {} |0.3(0+1.i)|\\= & {} |0.3i|\\= & {} 0.3. \end{aligned}$$

We would represent this CPFS set in matrix form as:

$$\begin{aligned}{}[{\mathcal {L}} ]_{3\times 3}= \begin{pmatrix} [0.2,0.5] &{} [0.1,0.8] &{} [0.6,0.8]\\ [0,0] &{} [0,0] &{} [0,0]\\ [0.5,0.7] &{} [0.4,0.6] &{} [0.8,0.3] \end{pmatrix}. \end{aligned}$$

#### Definition 3.1

Let $[ {\mathcal {L}} ]_{\hat{s}\times \breve{u}}$ be a CPFSM. Then $[ {\mathcal {L}} ]_{\hat{s}\times \breve{u}}$ is called complex Pythagorean fuzzy zero soft matrix if $[\varkappa _{ij}(\tilde{a}_{t}),\ell _{ij}(\tilde{a}_{t})]=[0,0]$ for all $[\varkappa _{ij}(\tilde{a}_{t}),\ell _{ij}(\tilde{a}_{t})]\in [{\mathcal {L}} ]_{\hat{s}\times \breve{u}}$ and denoted by $[ {\mathcal {L}} ]_{\hat{s}\times \breve{u}}=[0,0].$

#### Example 3.2

The matrix $$[ {\mathcal {L}} ]_{3\times 3}= \begin{pmatrix} [0,0] &{} [0,0] &{} [0,0]\\ [0,0] &{} [0,0] &{} [0,0]\\ [0,0] &{} [0,0] &{} [0,0] \end{pmatrix} $$ is a complex Pythagorean fuzzy zero soft matrix.

#### Definition 3.2

Let $[ {\mathcal {L}} ]_{\hat{s}\times \breve{u}}$ and $[{{\ss}} ]_{\hat{s}\times \breve{u}}$ be CPFSMs. Then

(*i*).: $[ {\mathcal {L}} ]_{\hat{s}\times \breve{u}}$ is a CPFS submatrices of $[{{\ss }}]_{\hat{s}\times \breve{u}}$, denoted by $[ {\mathcal {L}} ]_{\hat{s}\times \breve{u}}\subseteq [{{\ss }}]_{\hat{s}\times \breve{u}},$ if $\varkappa _{ij}(\tilde{a}_{t})\le \Re _{ij}(\tilde{a}_{t})$ and $\ell _{ij}(\tilde{a}_{t})\ge \eth _{ij}(\tilde{a}_{t})$ for all $[\varkappa _{ij}(\tilde{a}_{t}),\ell _{ij}(\tilde{a}_{t})]\in [{\mathcal {L}} ]_{\hat{s}\times \breve{u}}$ and $[\Re _{ij}(\tilde{a}_{t}),\eth _{ij}(\tilde{a}_{t})]\in [{{\ss }}]_{\hat{s}\times \breve{u}}.$
(*ii*).: $[ {\mathcal {L}} ]_{\hat{s}\times \breve{u}}$ is a proper CPFS submatrices of $[{{\ss }} ]_{\hat{s}\times \breve{u}},$ denoted by $[ {\mathcal {L}} ]_{\hat{s}\times \breve{u}}\subset [{{\ss }}]_{\hat{s}\times \breve{u} },$ if $\varkappa _{ij}(\tilde{a}_{t})\le \Re _{ij}(\tilde{a}_{t})$ and $\ell _{ij}(\tilde{a}_{t})\ge \eth _{ij}(\tilde{a}_{t})$ for all $[\varkappa _{ij}(\tilde{a}_{t}),\ell _{ij}(\tilde{a}_{t})]\in [{\mathcal {L}} ]_{\hat{s}\times \breve{u}}$ and $[\Re _{ij}(\tilde{a}_{t}),\eth _{ij}(\tilde{a}_{t})]\in [{{\ss }}]_{\hat{s}\times \breve{u}},$ and for at least one term $\varkappa _{ij}(\tilde{a}_{t})<\Re _{ij}(\tilde{a}_{t})$ and $\ell _{ij}(\tilde{a}_{t})>\eth _{ij}(\tilde{a}_{t}).$
(*iii*).: $[ {\mathcal {L}} ]_{\hat{s}\times \breve{u}}$ is an equal CPFSM of $[{{\ss }}]_{\hat{s} \times \breve{u}}$, denoted by $[ {\mathcal {L}} ]_{\hat{s}\times \breve{u}}=[{{\ss }}]_{\hat{s}\times \breve{u}}$ if $\varkappa _{ij}(\tilde{a}_{t})=\Re _{ij}(\tilde{a}_{t})$ and $\ell _{ij} (\tilde{a}_{t})=\eth _{ij}(\tilde{a}_{t})$ for all $[\varkappa _{ij}(\tilde{a}_{t}),\ell _{ij}(\tilde{a}_{t})]\in [{\mathcal {L}} ]_{\hat{s}\times \breve{u}}$ and $[\Re _{ij}(\tilde{a}_{t}),\eth _{ij}(\tilde{a}_{t})]\in [{{\ss }}]_{\hat{s}\times \breve{u}}.$

#### Definition 3.3

Let $[ {\mathcal {L}} ]_{\hat{s}\times \breve{u}}$ and $[{{\ss }}]_{\hat{s}\times \breve{u}}$ be CPFSMs. Then the union of $[ {\mathcal {L}} ]_{\hat{s}\times \breve{u}}$ and $[{{\ss }}]_{\hat{s}\times \breve{u}},$ denoted by $[ {\mathcal {L}} ]_{\hat{s}\times \breve{u}}\cup [{{\ss }}]_{\hat{s}\times \breve{u}}$ and defined as:

$$\begin{aligned}{}[{\mathcal {L}} ]_{\hat{s}\times \breve{u}}\cup [{{\ss }}]_{\hat{s}\times \breve{u}}=\{ \max [\varkappa _{ij}(\tilde{a}_{t}),\Re _{ij}(\tilde{a}_{t})],\min [\ell _{ij}(\tilde{a}_{t}),\eth _{ij}(\tilde{a}_{t})] \end{aligned}$$

for all $[\varkappa _{ij}(\tilde{a}_{t}),\ell _{ij}(\tilde{a}_{t})]\in [{\mathcal {L}} ]_{\hat{s}\times \breve{u}}$ and $[\Re _{ij}(\tilde{a}_{t}),\eth _{ij}(\tilde{a}_{t})]\in [{{\ss }}]_{\hat{s}\times \breve{u}}.$

#### Definition 3.4

Let $[ {\mathcal {L}} ]_{\hat{s}\times \breve{u}}$ and $[{{\ss }}]_{\hat{s}\times \breve{u}}$ be CPFSMs. Then the intersection of $[ {\mathcal {L}} ]_{\hat{s}\times \breve{u}}$ and $[{{\ss }}]_{\hat{s}\times \breve{u}},$ denoted by $[ {\mathcal {L}} ]_{\hat{s}\times \breve{u}}\cap [{{\ss }}]_{\hat{s}\times \breve{u}}$ and defined as:

$$\begin{aligned}{}[{\mathcal {L}} ]_{\hat{s}\times \breve{u}}\cap [{{\ss }}]_{\hat{s}\times \breve{u}}=\{ \min [\varkappa _{ij}(\tilde{a}_{t}),\Re _{ij}(\tilde{a}_{t})],\max [\ell _{ij}(\tilde{a}_{t}),\eth _{ij}(\tilde{a}_{t})] \end{aligned}$$

for all $[\varkappa _{ij}(\tilde{a}_{t}),\ell _{ij}(\tilde{a}_{t})]\in [{\mathcal {L}} ]_{\hat{s}\times \breve{u}}$ and $[\Re _{ij}(\tilde{a}_{t}),\eth _{ij}(\tilde{a}_{t})]\in [{{\ss }}]_{\hat{s}\times \breve{u}}.$

#### Definition 3.5

The complement of $[ {\mathcal {L}} ]_{\hat{s}\times \breve{u}}$ denoted by $[ {\mathcal {L}} ]_{\hat{s}\times \breve{u}}^{c}$ and defined as:

$$\begin{aligned}{}[{\mathcal {L}} ]_{\hat{s}\times \breve{u}}^{c}=[\ell _{ij}(\tilde{a}_{t}),\varkappa _{ij} (\tilde{a}_{t})] \end{aligned}$$

for all $$[\varkappa _{ij}(\tilde{a}_{t}),\ell _{ij}(\tilde{a}_{t})]\in [{\mathcal {L}} ]_{\hat{s}\times \breve{u}}.$$

#### Example 3.3

Assume that

$$\begin{aligned}{}[\grave{A}_{4\times 4}]&= \begin{bmatrix} [0.5,0.3] &{} [0.8,0.1] &{} [0.6,0.2] &{} [0.2,0.9]\\ [0.6,0.5] &{} [0.9,0] &{} [0,1] &{} [0.4,0.7]\\ [0.3,0.6] &{} [0.1,0.9] &{} [0.4,0.5] &{} [0.7,0.4]\\ [0.7,0.2] &{} [0,0.8] &{} [0.6,0.2] &{} [0.9,0] \end{bmatrix} ,\\ [{{\ss }}_{4\times 4}]&= \begin{bmatrix} [0.4,0.5] &{} [0.8,0.4] &{} [0,1] &{} [0.4,0.9]\\ [0.3,0.4] &{} [1,0] &{} [0.8,0.5] &{} [0.5,0.7]\\ [0.2,0.8] &{} [0.1,0.6] &{} [0.4,0.8] &{} [0.3,0.5]\\ [1,0] &{} [0.5,0.7] &{} [0.4,0.6] &{} [0.5,0.2] \end{bmatrix} . \end{aligned}$$

Then

$$\begin{aligned}{}[\grave{A}_{4\times 4}]\cup [{{\ss }}_{4\times 4}]= \begin{bmatrix} [0.5,0.3] &{} [0.8,0.1] &{} [0.6,0.2] &{} [0.4,0.9]\\ [0.6,0.4] &{} [0.9,0] &{} [0.8,0.5] &{} [0.5,0.7]\\ [0.3,0.6] &{} [0.1,0.6] &{} [0.4,0.5] &{} [0.7,0.4]\\ [1,0] &{} [0.5,0.7] &{} [0.6,0.2] &{} [0.9,0] \end{bmatrix} \end{aligned}$$

and

$$\begin{aligned}{}[\grave{A}_{4\times 4}]\cap [{{\ss }}_{4\times 4}]&= \begin{bmatrix} [0.4,0.5] &{} [0.8,0.4] &{} [0,1] &{} [0.2,0.9]\\ [0.3,0.5] &{} [0.9,0] &{} [0,1] &{} [0.4,0.7]\\ [0.2,0.8] &{} [0.1,0.9] &{} [0.4,0.8] &{} [0.3,0.5]\\ [0.7,0.2] &{} [0,0.8] &{} [0.4,0.6] &{} [0.5,0.2] \end{bmatrix} \\ [\grave{A}_{4\times 4}]^{c}&= \begin{bmatrix} [0.3,0.5] &{} [0.1,0.8] &{} [0.2,0.6] &{} [0.9,0.2]\\ [0.5,0.6] &{} [0,0.9] &{} [1,0] &{} [0.7,0.4]\\ [0.6,0.3] &{} [0.9,0.1] &{} [0.5,0.4] &{} [0.4,0.7]\\ [0.2,0.7] &{} [0.8,0] &{} [0.2,0.6] &{} [0,0.9] \end{bmatrix} . \end{aligned}$$

#### Definition 3.6

Let $[ {\mathcal {L}} ]_{\hat{s}\times \breve{u}}$ and $[{{\ss }}]_{\hat{s}\times \breve{u}}$ be CPFSMs. Then $[ {\mathcal {L}} ]_{\hat{s}\times \breve{u}}$ and $[{{\ss }}]_{\hat{s}\times \breve{u}}$ are disjoint if $[ {\mathcal {L}} ]_{\hat{s}\times \breve{u}}\cap [{{\ss }}]_{\hat{s}\times \breve{u} }=[0,0]$ for all $\hat{s}$ and $\breve{u}.$

#### Definition 3.7

Let $[ {\mathcal {L}} ]_{\hat{s}\times \breve{u}}$ and $[{{\ss }}]_{\hat{s}\times \breve{u}}$ be CPFSMs. Then, the difference between these two CPFSMs is defined by

$$\begin{aligned}{}[{\mathcal {L}} ]_{\hat{s}\times \breve{u}}\setminus [{{\ss }}]_{\hat{s}\times \breve{u}}=[\Re _{ij}-\varkappa _{ij},\eth _{ij}-\ell _{ij}] \end{aligned}$$

for all $[\varkappa _{ij}(\tilde{a}_{t}),\ell _{ij}(\tilde{a}_{t})]\in [{\mathcal {L}} ]_{\hat{s}\times \breve{u}}$ and $[\Re _{ij}(\tilde{a}_{t}),\eth _{ij}(\tilde{a}_{t})]\in [{{\ss }}]_{\hat{s}\times \breve{u}}.$

#### Definition 3.8

Let $[ {\mathcal {L}} ]_{\hat{s}\times \breve{u}}$ and $[{{\ss }}]_{\hat{s}\times \breve{u}}$ be CPFSMs. Then, the symmetric difference of these two CPFSMs denoted by $[ {\mathcal {L}} ]_{\hat{s}\times \breve{u}}\Delta [{{\ss }}]_{\hat{s}\times \breve{u}}$ and is defined as:

$$\begin{aligned}{}[{\mathcal {L}} ]_{\hat{s}\times \breve{u}}\Delta [{{\ss }}]_{\hat{s}\times \breve{u}}&=\{[ {\mathcal {L}} ]_{\hat{s}\times \breve{u}}\cup [{{\ss }}]_{\hat{s}\times \breve{u} }\}-\{[ {\mathcal {L}} ]_{\hat{s}\times \breve{u}}\cap [{{\ss }}]_{\hat{s}\times \breve{u}}\} \\&=[\max \{ \varkappa _{ij},\Re _{ij}\},\min \{ \ell _{ij},\eth _{ij}\}]-[\min \{ \varkappa _{ij},\Re _{ij}\},\max \{ \ell _{ij},\eth _{ij}\}] \end{aligned}$$

for all $[\varkappa _{ij}(\tilde{a}_{t}),\ell _{ij}(\tilde{a}_{t})]\in [{\mathcal {L}} ]_{\hat{s}\times \breve{u}}$ and $[\Re _{ij}(\tilde{a}_{t}),\eth _{ij}(\tilde{a}_{t})]\in [{{\ss }}]_{\hat{s}\times \breve{u}}.$

#### Definition 3.9

If we interchange the operation union ‘$\cup$’ and intersection ‘$\cap$’ to each other in any result for a CPFSM, we obtain the true result. Therefore, if any result is derivable from these operations, so is the true result is obtained by interchanging union by intersection and intersection by union. This is called the duality principle.

#### Example 3.4

Let

$$[\grave{A}_{2\times 2}]= \begin{pmatrix} [0.5,0.8] &{} [0.4,0.6]\\ [0.1,0.7] &{} [0.3,0.9] \end{pmatrix} $$ and [sss $$_{2\times 2}]= \begin{pmatrix} [0.8,0.3] &{} [0.6,0.5]\\ [0.4,0.8] &{} [0.2,0.7] \end{pmatrix} $$ then

$$\begin{aligned}{}[\grave{A}_{2\times 2}]\cup [{{\ss }}_{2\times 2}]&= \begin{pmatrix} [0.8,0.3] &{} [0.6,0.5]\\ [0.4,0.7] &{} [0.3,0.7] \end{pmatrix} \\ [{{\ss }}_{2\times 2}]\cup [\grave{A}_{2\times 2}]&= \begin{pmatrix} [0.8,0.3] &{} [0.6,0.5]\\ [0.4,0.7] &{} [0.3,0.7] \end{pmatrix} . \end{aligned}$$

Thus, the commutative law holds concerning the union. Now, to check the duality principle, we have to change the union by an intersection as follows:

$$\begin{aligned}{}[\grave{A}_{2\times 2}]\cap [{{\ss }}_{2\times 2}]&= \begin{pmatrix} [0.5,0.8] &{} [0.4,0.6]\\ [0.1,0.8] &{} [0.2,0.9] \end{pmatrix} \\ [{{\ss }}_{2\times 2}]\cap [\grave{A}_{2\times 2}]&= \begin{pmatrix} [0.5,0.8] &{} [0.4,0.6]\\ [0.1,0.8] &{} [0.2,0.9] \end{pmatrix} . \end{aligned}$$

Thus, the commutative law again holds. This means that by interchanging the We get the same derivable result by union by intersection and intersection by union.

### Ethical approval

This article does not contain any studies with human participants or animals performed by any of the authors.

## **Main results**

### Proposition 4.1

Let $[ {\mathcal {L}} ]_{\hat{s}\times \breve{u}}$ be a CPFS metric matrix then

(*i*): $$([ {\mathcal {L}} ]_{\hat{s}\times \breve{u}}^{\bullet })^{\bullet }=[ {\mathcal {L}} ]_{\hat{s}\times \breve{u}}.$$
(*ii*).: $$[0,0]^{\bullet }=[0,0].$$

### Proof

It can be proved easily. $\square$

### Proposition 4.2

Let $[ {\mathcal {L}} ]_{\hat{s}\times \breve{u}}$ and $[{{\ss }}]_{\hat{s}\times \breve{u}}$ be CPFSMs, then De-Margon Laws are valid.

(*i*).: $$([ {\mathcal {L}} ]_{\hat{s}\times \breve{u}}\cup [{{\ss }}]_{\hat{s}\times \breve{u} })^{\bullet }=[ {\mathcal {L}} ]_{\hat{s}\times \breve{u}}^{\bullet }\cap [{{\ss }}]_{\hat{s} \times \breve{u}}^{\bullet }.$$
(*ii*).: $$([ {\mathcal {L}} ]_{\hat{s}\times \breve{u}}\cap [{{\ss }}]_{\hat{s}\times \breve{u} })^{\bullet }=[ {\mathcal {L}} ]_{\hat{s}\times \breve{u}}^{\bullet }\cup [{{\ss }}]_{\hat{s} \times \breve{u}}^{\bullet }.$$

### Proof

(*i*).: For all $$\hat{s},$$ $$\breve{u}$$,
  $$\begin{aligned} ([ {\mathcal {L}} ]_{\hat{s}\times \breve{u}}\cup [{{\ss }}]_{\hat{s}\times \breve{u} })^{\bullet }=[\max \{ \varkappa _{ij},\Re _{ij}\},\min \{ \ell _{ij},\eth _{ij}\}]^{\bullet }. \end{aligned}$$
  For all $[\varkappa _{ij}(\tilde{a}_{t}),\ell _{ij}(\tilde{a}_{t})]\in [{\mathcal {L}} ]_{\hat{s}\times \breve{u}}$ and $[\Re _{ij}(\tilde{a}_{t}),\eth _{ij}(\tilde{a}_{t})]\in [{{\ss }}]_{\hat{s}\times \breve{u}}$ such that $\varkappa _{ij}\le \Re _{ij}$ and $\ell _{ij}\le \eth _{ij}$ then
  2 $$\begin{aligned} ([ {\mathcal {L}} ]_{\hat{s}\times \breve{u}}\cup [{{\ss }}]_{\hat{s}\times \breve{u} })^{\bullet }&=[\Re _{ij},\ell _{ij}]^{\bullet }\nonumber \\&=[\ell _{ij},\Re _{ij}] \end{aligned}$$
  also
  3 $$\begin{aligned}{}[{\mathcal {L}} ]_{\hat{s}\times \breve{u}}^{\bullet }\cap [{{\ss }}]_{\hat{s} \times \breve{u}}^{\bullet }&=[\min \{ \ell _{ij},\eth _{ij}\},\max \{ \varkappa _{ij},\Re _{ij}\}]\nonumber \\&=[\ell _{ij},\Re _{ij}]. \end{aligned}$$
  From (2) and (3), we have
  $$\begin{aligned} ([ {\mathcal {L}} ]_{\hat{s}\times \breve{u}}\cup [{{\ss }}]_{\hat{s}\times \breve{u} })^{\bullet }=[ {\mathcal {L}} ]_{\hat{s}\times \breve{u}}^{\bullet }\cap [{{\ss }}]_{\hat{s} \times \breve{u}}^{\bullet }. \end{aligned}$$
(*ii*).: By duality principle (*ii*) is also hold.

$$\square $$

### Proposition 4.3

If $[ {\mathcal {L}} ]_{\hat{s}\times \breve{u}},$ $[{{\ss }}]_{\hat{s}\times \breve{u}}$ and $[{\text {\Thorn}}]_{\hat{s}\times \breve{u}}$ are CPFSMs then

(*i*).: $$[ {\mathcal {L}} ]_{\hat{s}\times \breve{u}}=[{{\ss }}]_{\hat{s}\times \breve{u}}$$ and $$[{{\ss }}]_{\hat{s}\times \breve{u}}=[{\text {\Thorn}}]_{\hat{s}\times \breve{u} }\Longrightarrow [{\mathcal {L}} ]_{\hat{s}\times \breve{u}}=[{\text {\Thorn}}]_{\hat{s}\times \breve{u}}.$$
(*ii*).: $$[ {\mathcal {L}} ]_{\hat{s}\times \breve{u}}\subseteq [{{\ss }}]_{\hat{s}\times \breve{u}}$$ and $$[{{\ss }}]_{\hat{s}\times \breve{u}}\subseteq [{\mathcal {L}} ]_{\hat{s}\times \breve{u}}\Longleftrightarrow [{\mathcal {L}} ]_{\hat{s}\times \breve{u}}=[{{\ss }}]_{\hat{s}\times \breve{u}}.$$

### Proof

(*i*).: Assume that $[ {\mathcal {L}} ]_{\hat{s}\times \breve{u}}=[{{\ss }}]_{\hat{s}\times \breve{u}}$ and $[{{\ss }}]_{\hat{s}\times \breve{u}}=[{\text {\Thorn}}]_{\hat{s}\times \breve{u} }.$ Then $\varkappa _{ij}(\tilde{a}_{t})=\Re _{ij}(\tilde{a}_{t})$, $\ell _{ij}(\tilde{a}_{t})=\eth _{ij}(\tilde{a}_{t})$ and $\Re _{ij}(\tilde{a} _{t})=\sigma _{ij}^{\prime }(\tilde{a}_{t})$, $\eth _{ij}(\tilde{a}_{t} )=\sigma _{ij}^{\prime \prime }(\tilde{a}_{t})$ for all $[\varkappa _{ij} (\tilde{a}_{t}),\ell _{ij}(\tilde{a}_{t})]\in [{\mathcal {L}} ]_{\hat{s}\times \breve{u}}$, $[\Re _{ij}(\tilde{a}_{t}),\eth _{ij}(\tilde{a} _{t})]\in [{{\ss }}]_{\hat{s}\times \breve{u}}$ and $[\sigma _{ij}^{\prime }(\tilde{a}_{t}),\sigma _{ij}^{\prime \prime }(\tilde{a}_{t} )]\in [{\text {\Thorn}}]_{\hat{s}\times \breve{u}}.$ So by the transitive property, we have that $\varkappa _{ij}(\tilde{a}_{t})=\sigma _{ij}^{\prime }(\tilde{a} _{t})$ and $\ell _{ij}(\tilde{a}_{t})=\sigma _{ij}^{\prime \prime }(\tilde{a} _{t})$. This implies that $[ {\mathcal {L}} ]_{\hat{s}\times \breve{u}}=[{\text {\Thorn}}]_{\hat{s}\times \breve{u}}.$
(*ii*).: Assume that $[ {\mathcal {L}} ]_{\hat{s}\times \breve{u}}\subseteq [{{\ss }}]_{\hat{s}\times \breve{u}}.$ Then $\varkappa _{ij}(\tilde{a}_{t})\le \Re _{ij}(\tilde{a}_{t})$ and $\ell _{ij}(\tilde{a}_{t})\ge \eth _{ij}(\tilde{a}_{t}).$ Also $[{{\ss }} ]_{\hat{s}\times \breve{u}}\subseteq [{\mathcal {L}} ]_{\hat{s}\times \breve{u}}$ implies that $\Re _{ij}(\tilde{a}_{t})\le \varkappa _{ij}(\tilde{a}_{t})$ and $\eth _{ij}(\tilde{a}_{t})\ge \ell _{ij}(\tilde{a}_{t}).$ Thus we have $\varkappa _{ij}(\tilde{a}_{t})=\Re _{ij}(\tilde{a}_{t})$ and $\ell _{ij}(\tilde{a}_{t})=\eth _{ij}(\tilde{a}_{t})$ and hence $[ {\mathcal {L}} ]_{\hat{s}\times \breve{u}}=[{{\ss }}]_{\hat{s}\times \breve{u}}.$ Conversely, assume that $[ {\mathcal {L}} ]_{\hat{s}\times \breve{u}}=[{{\ss }}]_{\hat{s}\times \breve{u}}.$ Then for all $[\varkappa _{ij}(\tilde{a}_{t}),\ell _{ij}(\tilde{a}_{t})]\in [{\mathcal {L}} ]_{\hat{s}\times \breve{u}}$, $[\Re _{ij}(\tilde{a}_{t}),\eth _{ij}(\tilde{a} _{t})]\in [{{\ss }}]_{\hat{s}\times \breve{u}},$ we have $\varkappa _{ij}(\tilde{a}_{t})=\Re _{ij}(\tilde{a}_{t})$ and $\ell _{ij}(\tilde{a} _{t})=\eth _{ij}(\tilde{a}_{t}).$ This implies that $\varkappa _{ij}(\tilde{a}_{t})\le \Re _{ij}(\tilde{a}_{t})$ and $\ell _{ij}(\tilde{a}_{t})\ge \eth _{ij}(\tilde{a}_{t})$ and also $\Re _{ij}(\tilde{a}_{t})\le \varkappa _{ij}(\tilde{a}_{t})$ and $\eth _{ij}(\tilde{a}_{t})\ge \ell _{ij}(\tilde{a} _{t}).$ Thus $[ {\mathcal {L}} ]_{\hat{s}\times \breve{u}}\subseteq [{{\ss }}]_{\hat{s}\times \breve{u}}$ and $[{{\ss }}]_{\hat{s}\times \breve{u}}\subseteq [{\mathcal {L}} ]_{\hat{s}\times \breve{u}}.$

$$\square $$

### Proposition 4.4

If $[ {\mathcal {L}} ]_{\hat{s}\times \breve{u}},$ $[{{\ss }}]_{\hat{s}\times \breve{u}}$ and $[{\text {\Thorn}}]_{\hat{s}\times \breve{u}}$ are CPFSMs then

(*i*).: $$[ {\mathcal {L}} ]_{\hat{s}\times \breve{u}}\subseteq [{{\ss }}]_{\hat{s}\times \breve{u}}$$ and $$[{{\ss }}]_{\hat{s}\times \breve{u}}\subseteq [{\text {\Thorn}}]_{\hat{s}\times \breve{u}}\Longrightarrow [{\mathcal {L}} ]_{\hat{s}\times \breve{u}}\subseteq [{\text {\Thorn}}]_{\hat{s}\times \breve{u}}.$$
(*ii*).: $$[ {\mathcal {L}} ]_{\hat{s}\times \breve{u}}\subseteq [{{\ss }}]_{\hat{s}\times \breve{u}}\Longleftrightarrow [{\mathcal {L}} ]_{\hat{s}\times \breve{u}}\cap [{{\ss }}]_{\hat{s}\times \breve{u}}=[ {\mathcal {L}} ]_{\hat{s}\times \breve{u}}.$$
(*iii*).: $$[ {\mathcal {L}} ]_{\hat{s}\times \breve{u}}\subseteq [{{\ss }}]_{\hat{s}\times \breve{u}}\Longleftrightarrow [{\mathcal {L}} ]_{\hat{s}\times \breve{u}}\cup [{{\ss }}]_{\hat{s}\times \breve{u} }=[{{\ss }}]_{\hat{s}\times \breve{u}}.$$

### Proof

(*i*).: Assume that $[ {\mathcal {L}} ]_{\hat{s}\times \breve{u}}\subseteq [{{\ss }}]_{\hat{s}\times \breve{u}}$ and $[{{\ss }}]_{\hat{s}\times \breve{u}}\subseteq [{\text {\Thorn}}]_{\hat{s}\times \breve{u}}.$ Then $\varkappa _{ij}(\tilde{a} _{t})\le \eth _{ij}(\tilde{a}_{t})$, $\ell _{ij}(\tilde{a}_{t})\ge \eth _{ij}(\tilde{a}_{t})$ and $\Re _{ij}(\tilde{a}_{t})\le \sigma _{ij}^{\prime }(\tilde{a}_{t})$, $\eth _{ij}(\tilde{a}_{t})\ge \sigma _{ij}^{\prime \prime }(\tilde{a}_{t})$ for all $[\varkappa _{ij}(\tilde{a}_{t}),\ell _{ij}(\tilde{a}_{t})]\in [{\mathcal {L}} ]_{\hat{s}\times \breve{u}}$, $[\Re _{ij}(\tilde{a}_{t}),\eth _{ij}(\tilde{a} _{t})]\in [{{\ss }}]_{\hat{s}\times \breve{u}}$ and $[\sigma _{ij}^{\prime },\sigma _{ij}^{\prime \prime }]\in [{\text {\Thorn}}]_{\hat{s}\times \breve{u}}.$ So by the transitive property, we have that $\varkappa _{ij}(\tilde{a}_{t})\le \sigma _{ij}^{\prime }(\tilde{a}_{t})$ and $\ell _{ij}(\tilde{a}_{t})\ge \sigma _{ij}^{\prime \prime }(\tilde{a}_{t})$. This implies that $[ {\mathcal {L}} ]_{\hat{s}\times \breve{u}}\subseteq [{\text {\Thorn}}]_{\hat{s}\times \breve{u}}.$
(*ii*).: Assume that $[ {\mathcal {L}} ]_{\hat{s}\times \breve{u}}\subseteq [{{\ss }}]_{\hat{s}\times \breve{u}}.$ Then for all $[\varkappa _{ij}(\tilde{a}_{t}),\ell _{ij}(\tilde{a} _{t})]\in [{\mathcal {L}} ]_{\hat{s}\times \breve{u}}$, $[\Re _{ij}(\tilde{a}_{t}),\eth _{ij}(\tilde{a} _{t})]\in [{{\ss }}]_{\hat{s}\times \breve{u}},$ we have $\varkappa _{ij}(\tilde{a}_{t})\le \Re _{ij}(\tilde{a}_{t})$ and $\ell _{ij}(\tilde{a} _{t})\ge \eth _{ij}(\tilde{a}_{t}).$ Now
  $$\begin{aligned}{}[{\mathcal {L}} ]_{\hat{s}\times \breve{u}}\cap [{{\ss }}]_{\hat{s}\times \breve{u}}&=[\min \{ \varkappa _{ij}(\tilde{a}_{t}),\Re _{ij}(\tilde{a}_{t})\},\max \{ \ell _{ij}(\tilde{a}_{t}),\eth _{ij}(\tilde{a}_{t})\} \\&=[\varkappa _{ij}(\tilde{a}_{t}),\ell _{ij}(\tilde{a}_{t})]=[ {\mathcal {L}} ]_{\hat{s}\times \breve{u}}. \end{aligned}$$
  Conversely, assume that $[ {\mathcal {L}} ]_{\hat{s}\times \breve{u}}\cap [{{\ss }}]_{\hat{s}\times \breve{u}}=[ {\mathcal {L}} ]_{\hat{s}\times \breve{u}}.$ This implies that $\varkappa _{ij}(\tilde{a} _{t})\le \Re _{ij}(\tilde{a}_{t})$ and $\ell _{ij}(\tilde{a}_{t})\ge \eth _{ij}(\tilde{a}_{t})$ for all $[\varkappa _{ij}(\tilde{a}_{t}),\ell _{ij} (\tilde{a}_{t})]\in [{\mathcal {L}} ]_{\hat{s}\times \breve{u}}$ and $[\Re _{ij}(\tilde{a}_{t}),\eth _{ij}(\tilde{a}_{t})]\in [{{\ss }}]_{\hat{s}\times \breve{u}}.$ Hence $[ {\mathcal {L}} ]_{\hat{s}\times \breve{u}}\subseteq [{{\ss }}]_{\hat{s}\times \breve{u}}.$
(*iii*).: Assume that $[ {\mathcal {L}} ]_{\hat{s}\times \breve{u}}\subseteq [{{\ss }}]_{\hat{s}\times \breve{u}}.$ Then for all $[\varkappa _{ij}(\tilde{a}_{t}),\ell _{ij}(\tilde{a} _{t})]\in [{\mathcal {L}} ]_{\hat{s}\times \breve{u}}$ and $[\Re _{ij}(\tilde{a}_{t}),\eth _{ij}(\tilde{a}_{t})]\in [{{\ss }}]_{\hat{s}\times \breve{u}},$ we have $\varkappa _{ij}(\tilde{a}_{t})\le \Re _{ij}(\tilde{a}_{t})$ and $\ell _{ij}(\tilde{a}_{t})\ge \eth _{ij}(\tilde{a}_{t}).$ Now
  $$\begin{aligned}{}[{\mathcal {L}} ]_{\hat{s}\times \breve{u}}\cup [{{\ss }}]_{\hat{s}\times \breve{u}}&=[\max \{ \varkappa _{ij}(\tilde{a}_{t}),\Re _{ij}(\tilde{a}_{t})\},\min \{ \ell _{ij}(\tilde{a}_{t}),\eth _{ij}(\tilde{a}_{t})\} \\&=[\Re _{ij}(\tilde{a}_{t}),\eth _{ij}(\tilde{a}_{t})]=[{{\ss }}]_{\hat{s}\times \breve{u}}. \end{aligned}$$
  Conversely, assume that $[ {\mathcal {L}} ]_{\hat{s}\times \breve{u}}\cup [{{\ss }}]_{\hat{s}\times \breve{u} }=[{{\ss }}]_{\hat{s}\times \breve{u}}.$ This implies that $\varkappa _{ij}(\tilde{a}_{t})\le \Re _{ij}(\tilde{a}_{t})$ and $\ell _{ij}(\tilde{a} _{t})\ge \eth _{ij}(\tilde{a}_{t})$ for all $[\varkappa _{ij}(\tilde{a} _{t}),\ell _{ij}(\tilde{a}_{t})]\in [{\mathcal {L}} ]_{\hat{s}\times \breve{u}}$ and $[\Re _{ij}(\tilde{a}_{t}),\eth _{ij}(\tilde{a}_{t})]\in [{{\ss }}]_{\hat{s}\times \breve{u}}.$ Hence $[ {\mathcal {L}} ]_{\hat{s}\times \breve{u}}\subseteq [{{\ss }}]_{\hat{s}\times \breve{u}}.$

$$\square $$

### Proposition 4.5

If $[ {\mathcal {L}} ]_{\hat{s}\times \breve{u}}$ and $[{{\ss }}]_{\hat{s}\times \breve{u}}$ are CPFSMs then

(*i*).: $$[ {\mathcal {L}} ]_{\hat{s}\times \breve{u}}\cup [{{\ss }}]_{\hat{s}\times \breve{u} }=[{{\ss }}]_{\hat{s}\times \breve{u}}\cup [{\mathcal {L}} ]_{\hat{s}\times \breve{u}}.$$
(*ii*).: $$[ {\mathcal {L}} ]_{\hat{s}\times \breve{u}}\cap [{{\ss }}]_{\hat{s}\times \breve{u} }=[{{\ss }}]_{\hat{s}\times \breve{u}}\cap [{\mathcal {L}} ]_{\hat{s}\times \breve{u}}.$$

### Proof

To prove (*i*),  we take two cases here. $\square$

**Case 1.** For all $[\varkappa _{ij}(\tilde{a}_{t}),\ell _{ij}(\tilde{a}_{t})]\in [{\mathcal {L}} ]_{\hat{s}\times \breve{u}}$ and $[\Re _{ij}(\tilde{a}_{t}),\eth _{ij}(\tilde{a}_{t})]\in [{{\ss }}]_{\hat{s}\times \breve{u}}$ such that $\varkappa _{ij}(\tilde{a}_{t})\le \Re _{ij}(\tilde{a}_{t})$ and $\ell _{ij}(\tilde{a}_{t})\le \eth _{ij}(\tilde{a}_{t})$ then

4 $$\begin{aligned}{}[{\mathcal {L}} ]_{\hat{s}\times \breve{u}}\cup [{{\ss }}]_{\hat{s}\times \breve{u}}&=[\max \{ \varkappa _{ij}(\tilde{a}_{t}),\Re _{ij}(\tilde{a}_{t})\},\min \{ \ell _{ij}(\tilde{a}_{t}),\eth _{ij}(\tilde{a}_{t})\}]\nonumber \\&=[\Re _{ij}(\tilde{a}_{t}),\ell _{ij}(\tilde{a}_{t})]. \end{aligned}$$

Also,

5 $$\begin{aligned}{}[{{\ss }}]_{\hat{s}\times \breve{u}}\cup [{\mathcal {L}} ]_{\hat{s}\times \breve{u}}&=[\max \{ \Re _{ij}(\tilde{a}_{t}),\varkappa _{ij}(\tilde{a}_{t})\},\min \{ \eth _{ij}(\tilde{a}_{t}),\ell _{ij}(\tilde{a} _{t})\}]\nonumber \\&=[\Re _{ij}(\tilde{a}_{t}),\ell _{ij}(\tilde{a}_{t})]. \end{aligned}$$

From (4) and (5), we have

$$\begin{aligned}{}[{\mathcal {L}} ]_{\hat{s}\times \breve{u}}\cup [{{\ss }}]_{\hat{s}\times \breve{u} }=[{{\ss }}]_{\hat{s}\times \breve{u}}\cup [{\mathcal {L}} ]_{\hat{s}\times \breve{u}}. \end{aligned}$$

**Case 2.** If $\varkappa _{ij}(\tilde{a}_{t})\ge \Re _{ij}(\tilde{a}_{t})$ and $\ell _{ij}(\tilde{a}_{t})\ge \eth _{ij}(\tilde{a}_{t})$ for all $[\varkappa _{ij}(\tilde{a}_{t}),\ell _{ij}(\tilde{a}_{t})]\in [{\mathcal {L}} ]_{\hat{s}\times \breve{u}}$ and $[\Re _{ij}(\tilde{a}_{t}),\eth _{ij}(\tilde{a}_{t})]\in [{{\ss }}]_{\hat{s}\times \breve{u}}$ then

6 $$\begin{aligned}{}[{\mathcal {L}} ]_{\hat{s}\times \breve{u}}\cup [{{\ss }}]_{\hat{s}\times \breve{u}}&=[\max \{ \varkappa _{ij}(\tilde{a}_{t}),\Re _{ij}(\tilde{a}_{t})\},\min \{ \ell _{ij}(\tilde{a}_{t}),\eth _{ij}(\tilde{a}_{t})\}]\nonumber \\&=[\varkappa _{ij}(\tilde{a}_{t}),\eth _{ij}(\tilde{a}_{t})] \end{aligned}$$

Also

7 $$\begin{aligned}{}[{{\ss }}]_{\hat{s}\times \breve{u}}\cup [{\mathcal {L}} ]_{\hat{s}\times \breve{u}}&=[\max \{ \Re _{ij}(\tilde{a}_{t}),\varkappa _{ij}(\tilde{a}_{t})\},\min \{ \eth _{ij}(\tilde{a}_{t}),\ell _{ij}(\tilde{a} _{t})\}]\nonumber \\&=[\varkappa _{ij}(\tilde{a}_{t}),\eth _{ij}(\tilde{a}_{t})] \end{aligned}$$

From (6) and (7), we have

$$\begin{aligned}{}[\tilde{a}_{\hat{s}\times \breve{u}}]\cup [Y_{\hat{s}\times \breve{u}}]=[Y_{\hat{s}\times \breve{u}}]\cup [\tilde{a}_{\hat{s} \times \breve{u}}]. \end{aligned}$$

Thus, in all cases, the commutative law of union holds. (*ii*). By duality principle (*ii*) is also hold.

### Theorem 4.6

If $[ {\mathcal {L}} ]_{\hat{s}\times \breve{u}},$ $[{{\ss }}]_{\hat{s}\times \breve{u}}$ and $[{\text {\Thorn}}]_{\hat{s}\times \breve{u}}$ are CPFSMs then

(*i*).: $$([ {\mathcal {L}} ]_{\hat{s}\times \breve{u}}\cup [{{\ss }}]_{\hat{s}\times \breve{u} })\cup [{\text {\Thorn}}]_{\hat{s}\times \breve{u}}=[ {\mathcal {L}} ]_{\hat{s}\times \breve{u}}\cup ([{{\ss }}]_{\hat{s}\times \breve{u}} \cup [{\text {\Thorn}}]_{\hat{s}\times \breve{u}}).$$
(*ii*).: $$([ {\mathcal {L}} ]_{\hat{s}\times \breve{u}}\cap [{{\ss }}]_{\hat{s}\times \breve{u} })\cap [{\text {\Thorn}}]_{\hat{s}\times \breve{u}}=[ {\mathcal {L}} ]_{\hat{s}\times \breve{u}}\cap ([{{\ss }}]_{\hat{s}\times \breve{u}} \cap [{\text {\Thorn}}]_{\hat{s}\times \breve{u}}).$$

### Proof

To prove (*i*),  we take three cases here. $\square$

*Case 1.* For all $[\varkappa _{ij}(\tilde{a}_{t}),\ell _{ij}(\tilde{a}_{t})]\in [{\mathcal {L}} ]_{\hat{s}\times \breve{u}}$, $[\phi _{ij}^{\prime }(\tilde{a}_{t}),\phi _{ij}^{\prime \prime }(\tilde{a}_{t})]\in [{{\ss }}]_{\hat{s} \times \breve{u}}$ and $[\Re _{ij}(\tilde{a}_{t}),\eth _{ij}(\tilde{a}_{t} )]\in [{\text {\Thorn}}]_{\hat{s}\times \breve{u}}$ such that $\varkappa _{ij}(\tilde{a}_{t})\le \phi _{ij}^{\prime }(\tilde{a}_{t})\le \Re _{ij} (\tilde{a}_{t})$ and $\ell _{ij}(\tilde{a}_{t})\le \phi _{ij}^{\prime \prime }(\tilde{a}_{t})\le \eth _{ij}(\tilde{a}_{t})$ then

8 $$\begin{aligned} ([ {\mathcal {L}} ]_{\hat{s}\times \breve{u}}\cup [{{\ss }}]_{\hat{s}\times \breve{u} })\cup [{\text {\Thorn}}]_{\hat{s}\times \breve{u}}&=[\max \{ \varkappa _{ij}(\tilde{a}_{t}),\phi _{ij}^{\prime }(\tilde{a}_{t})\},\min \{ \ell _{ij}(\tilde{a}_{t}),\phi _{ij}^{\prime \prime }(\tilde{a}_{t})\}] \cup [{\text {\Thorn}}]_{\hat{s}\times \breve{u}}\nonumber \\&=[\phi _{ij}^{\prime }(\tilde{a}_{t}),\ell _{ij}(\tilde{a}_{t})]\cup [\Re _{ij}(\tilde{a}_{t}),\eth _{ij}(\tilde{a}_{t})]\nonumber \\&=[\max \{ \phi _{ij}^{\prime }(\tilde{a}_{t}),\Re _{ij}(\tilde{a}_{t})\},\min \{ \ell _{ij}(\tilde{a}_{t}),\eth _{ij}(\tilde{a}_{t})\}]\nonumber \\&=[\Re _{ij}(\tilde{a}_{t}),\ell _{ij}(\tilde{a}_{t})]. \end{aligned}$$

9 $$\begin{aligned}{}[{\mathcal {L}} ]_{\hat{s}\times \breve{u}}\cup ([{{\ss }}]_{\hat{s}\times \breve{u}} \cup [{\text {\Thorn}}]_{\hat{s}\times \breve{u}})&=[ {\mathcal {L}} ]_{\hat{s}\times \breve{u}}\cup [\max \{ \phi _{ij}^{\prime }(\tilde{a} _{t}),\Re _{ij}(\tilde{a}_{t})\}, \min \{ \phi _{ij}^{\prime \prime }(\tilde{a}_{t}),\eth _{ij}(\tilde{a} _{t})\}]\nonumber \\&=[\varkappa _{ij}(\tilde{a}_{t}),\ell _{ij}(\tilde{a}_{t})]\cup [\Re _{ij}(\tilde{a}_{t}),\phi _{ij}^{\prime \prime }(\tilde{a}_{t})]\nonumber \\&=[\max \{ \varkappa _{ij}(\tilde{a}_{t}),\Re _{ij}(\tilde{a}_{t})\},\min \{ \ell _{ij}(\tilde{a}_{t}),\phi _{ij}^{\prime \prime }(\tilde{a}_{t})\}]\nonumber \\&=[\Re _{ij}(\tilde{a}_{t}),\ell _{ij}(\tilde{a}_{t})]. \end{aligned}$$

From (8) and (9), we have

$$\begin{aligned} ([ {\mathcal {L}} ]_{\hat{s}\times \breve{u}}\cup [{{\ss }}]_{\hat{s}\times \breve{u} })\cup [{\text {\Thorn}}]_{\hat{s}\times \breve{u}}=[ {\mathcal {L}} ]_{\hat{s}\times \breve{u}}\cup ([{{\ss }}]_{\hat{s}\times \breve{u}} \cup [{\text {\Thorn}}]_{\hat{s}\times \breve{u}}). \end{aligned}$$

*Case 2.* For all $[\varkappa _{ij}(\tilde{a}_{t}),\ell _{ij}(\tilde{a}_{t})]\in [{\mathcal {L}} ]_{\hat{s}\times \breve{u}}$, $[\phi _{ij}^{\prime }(\tilde{a}_{t}),\phi _{ij}^{\prime \prime }(\tilde{a}_{t})]\in [{{\ss }}]_{\hat{s} \times \breve{u}}$ and $[\Re _{ij}(\tilde{a}_{t}),\eth _{ij}(\tilde{a}_{t} )]\in [{\text {\Thorn}}]_{\hat{s}\times \breve{u}}$ such that $\varkappa _{ij}(\tilde{a}_{t})\le \Re _{ij}(\tilde{a}_{t})\le \phi _{ij}^{\prime } (\tilde{a}_{t})$ and $\ell _{ij}(\tilde{a}_{t})\le \eth _{ij}(\tilde{a}_{t} )\le \phi _{ij}^{\prime \prime }(\tilde{a}_{t})$ then

10 $$\begin{aligned} ([ {\mathcal {L}} ]_{\hat{s}\times \breve{u}}\cup [{{\ss }}]_{\hat{s}\times \breve{u} })\cup [{\text {\Thorn}}]_{\hat{s}\times \breve{u}}&=[\max \{ \varkappa _{ij}(\tilde{a}_{t}),\phi _{ij}^{\prime }(\tilde{a}_{t})\},\min \{ \ell _{ij}(\tilde{a}_{t}),\phi _{ij}^{\prime \prime }(\tilde{a}_{t})\}] \cup [{\text {\Thorn}}]_{\hat{s}\times \breve{u}}\nonumber \\&=[\phi _{ij}^{\prime }(\tilde{a}_{t}),\ell _{ij}(\tilde{a}_{t})]\cup [\Re _{ij}(\tilde{a}_{t}),\eth _{ij}(\tilde{a}_{t})]\nonumber \\&=[\max \{ \phi _{ij}^{\prime }(\tilde{a}_{t}),\Re _{ij}(\tilde{a}_{t})\},\min \{ \ell _{ij}(\tilde{a}_{t}),\eth _{ij}(\tilde{a}_{t})\}]\nonumber \\&=[\phi _{ij}^{\prime }(\tilde{a}_{t}),\ell _{ij}(\tilde{a}_{t})] \end{aligned}$$

11 $$\begin{aligned}{}[{\mathcal {L}} ]_{\hat{s}\times \breve{u}}\cup ([{{\ss }}]_{\hat{s}\times \breve{u}} \cup [{\text {\Thorn}}]_{\hat{s}\times \breve{u}})&=[ {\mathcal {L}} ]_{\hat{s}\times \breve{u}}\cup [\max \{ \phi _{ij}^{\prime }(\tilde{a} _{t}),\Re _{ij}(\tilde{a}_{t})\}, \min \{ \phi _{ij}^{\prime \prime }(\tilde{a}_{t}),\eth _{ij}(\tilde{a} _{t})\}]\nonumber \\&=[\varkappa _{ij}(\tilde{a}_{t}),\ell _{ij}(\tilde{a}_{t})]\cup [\phi _{ij}^{\prime }(\tilde{a}_{t}),\eth _{ij}(\tilde{a}_{t})]\nonumber \\&=[\max \{ \varkappa _{ij}(\tilde{a}_{t}),\phi _{ij}^{\prime }(\tilde{a} _{t})\},\min \{ \ell _{ij}(\tilde{a}_{t}),\eth _{ij}(\tilde{a}_{t})\}]\nonumber \\&=[\phi _{ij}^{\prime }(\tilde{a}_{t}),\ell _{ij}(\tilde{a}_{t})]. \end{aligned}$$

Equations (10) and (11), implies that

$$\begin{aligned} ([ {\mathcal {L}} ]_{\hat{s}\times \breve{u}}\cup [{{\ss }}]_{\hat{s}\times \breve{u} })\cup [{\text {\Thorn}}]_{\hat{s}\times \breve{u}}=[ {\mathcal {L}} ]_{\hat{s}\times \breve{u}}\cup ([{{\ss }}]_{\hat{s}\times \breve{u}} \cup [{\text {\Thorn}}]_{\hat{s}\times \breve{u}}). \end{aligned}$$

*Case 3.* For all $[\varkappa _{ij}(\tilde{a}_{t}),\ell _{ij}(\tilde{a}_{t})]\in [{\mathcal {L}} ]_{\hat{s}\times \breve{u}}$, $[\phi _{ij}^{\prime }(\tilde{a}_{t}),\phi _{ij}^{\prime \prime }(\tilde{a}_{t})]\in [{{\ss }}]_{\hat{s} \times \breve{u}}$ and $[\Re _{ij}(\tilde{a}_{t}),\eth _{ij}(\tilde{a}_{t} )]\in [{\text {\Thorn}}]_{\hat{s}\times \breve{u}}$ such that $\Re _{ij} (\tilde{a}_{t})\le \phi _{ij}^{\prime }(\tilde{a}_{t})\le \varkappa _{ij} (\tilde{a}_{t})$and $\eth _{ij}(\tilde{a}_{t})\le \phi _{ij}^{\prime \prime }(\tilde{a}_{t})\le \ell _{ij}(\tilde{a}_{t})$ then

12 $$\begin{aligned} ([ {\mathcal {L}} ]_{\hat{s}\times \breve{u}}\cup [{{\ss }}]_{\hat{s}\times \breve{u} })\cup [{\text {\Thorn}}]_{\hat{s}\times \breve{u}}&=[\max \{ \varkappa _{ij}(\tilde{a}_{t}),\phi _{ij}^{\prime }(\tilde{a}_{t})\},\min \{ \ell _{ij}(\tilde{a}_{t}),\phi _{ij}^{\prime \prime }(\tilde{a}_{t})\}] \cup [{\text {\Thorn}}]_{\hat{s}\times \breve{u}}\nonumber \\&=[\varkappa _{ij}(\tilde{a}_{t}),\phi _{ij}^{\prime \prime }(\tilde{a} _{t})]\cup [\Re _{ij}(\tilde{a}_{t}),\eth _{ij}(\tilde{a}_{t})]\nonumber \\&=[\max \{ \varkappa _{ij}(\tilde{a}_{t}),\Re _{ij}(\tilde{a}_{t})\},\min \{ \phi _{ij}^{\prime \prime }(\tilde{a}_{t}),\eth _{ij}(\tilde{a}_{t})\}]\nonumber \\&=[\varkappa _{ij}(\tilde{a}_{t}),\eth _{ij}(\tilde{a}_{t})]. \end{aligned}$$

13 $$\begin{aligned}{}[{\mathcal {L}} ]_{\hat{s}\times \breve{u}}\cup ([{{\ss }}]_{\hat{s}\times \breve{u}} \cup [{\text {\Thorn}}]_{\hat{s}\times \breve{u}})&=[ {\mathcal {L}} ]_{\hat{s}\times \breve{u}}\cup [\max \{ \phi _{ij}^{\prime }(\tilde{a} _{t}),\Re _{ij}(\tilde{a}_{t})\}, \min \{ \phi _{ij}^{\prime \prime }(\tilde{a}_{t}),\eth _{ij}(\tilde{a} _{t})\}]\nonumber \\&=[\varkappa _{ij}(\tilde{a}_{t}),\ell _{ij}(\tilde{a}_{t})]\cup [\phi _{ij}^{\prime }(\tilde{a}_{t}),\eth _{ij}(\tilde{a}_{t})]\nonumber \\&=[\max \{ \varkappa _{ij}(\tilde{a}_{t}),\phi _{ij}^{\prime }(\tilde{a} _{t})\},\min \{ \ell _{ij}(\tilde{a}_{t}),\eth _{ij}(\tilde{a}_{t})\}]\nonumber \\&=[\varkappa _{ij}(\tilde{a}_{t}),\eth _{ij}(\tilde{a}_{t})] \end{aligned}$$

Equations (12) and (13) implies that

$$\begin{aligned} ([ {\mathcal {L}} ]_{\hat{s}\times \breve{u}}\cup [{{\ss }}]_{\hat{s}\times \breve{u} })\cup [{\text {\Thorn}}]_{\hat{s}\times \breve{u}}=[ {\mathcal {L}} ]_{\hat{s}\times \breve{u}}\cup ([{{\ss }}]_{\hat{s}\times \breve{u}} \cup [{\text {\Thorn}}]_{\hat{s}\times \breve{u}}). \end{aligned}$$

Thus, in all cases, the associative law of union holds.

(*ii*). By duality principle, (*ii*) is also hold.

### Theorem 4.7

If $[ {\mathcal {L}} ]_{\hat{s}\times \breve{u}},$ $[{{\ss }}]_{\hat{s}\times \breve{u}}$ and $[{\text {\Thorn}}]_{\hat{s}\times \breve{u}}$ are CPFSMs then

(*i*).: $$[ {\mathcal {L}} ]_{\hat{s}\times \breve{u}}\cup ([{{\ss }}]_{\hat{s}\times \breve{u}} \cap [{\text {\Thorn}}]_{\hat{s}\times \breve{u}})=([ {\mathcal {L}} ]_{\hat{s}\times \breve{u}}\cup [{{\ss }}]_{\hat{s}\times \breve{u} })\cap ([ {\mathcal {L}} ]_{\hat{s}\times \breve{u}}\cup [{\text {\Thorn}}]_{\hat{s}\times \breve{u}}).$$
(*ii*).: $$[ {\mathcal {L}} ]_{\hat{s}\times \breve{u}}\cap ([{{\ss }}]_{\hat{s}\times \breve{u}} \cup [{\text {\Thorn}}]_{\hat{s}\times \breve{u}})=([ {\mathcal {L}} ]_{\hat{s}\times \breve{u}}\cap ([{{\ss }}]_{\hat{s}\times \breve{u}})\cup ([ {\mathcal {L}} ]_{\hat{s}\times \breve{u}}\cap [{\text {\Thorn}}]_{\hat{s}\times \breve{u}}).$$

### Proof

To prove (*i*),  we take three cases here. $\square$

*Case 1.* For all $[\varkappa _{ij}(\tilde{a}_{t}),\ell _{ij}(\tilde{a}_{t})]\in [{\mathcal {L}} ]_{\hat{s}\times \breve{u}}$, $[\phi _{ij}^{\prime }(\tilde{a}_{t}),\phi _{ij}^{\prime \prime }(\tilde{a}_{t})]\in [{{\ss }}]_{\hat{s} \times \breve{u}}$ and $[\Re _{ij}(\tilde{a}_{t}),\eth _{ij}(\tilde{a}_{t} )]\in [{\text {\Thorn}}]_{\hat{s}\times \breve{u}}$ such that $\varkappa _{ij}(\tilde{a}_{t})\le \phi _{ij}^{\prime }(\tilde{a}_{t})\le \Re _{ij} (\tilde{a}_{t})$ and $\ell _{ij}(\tilde{a}_{t})\le \phi _{ij}^{\prime \prime }(\tilde{a}_{t})\le \eth _{ij}(\tilde{a}_{t})$ then

14 $$\begin{aligned}{}[{\mathcal {L}} ]_{\hat{s}\times \breve{u}}\cup ([{{\ss }}]_{\hat{s}\times \breve{u}} \cap [{\text {\Thorn}}]_{\hat{s}\times \breve{u}})&=[ {\mathcal {L}} ]_{\hat{s}\times \breve{u}}\cup [\min \{ \phi _{ij}^{\prime }(\tilde{a} _{t}),\Re _{ij}(\tilde{a}_{t})\}, \max \{ \phi _{ij}^{\prime \prime }(\tilde{a}_{t}),\eth _{ij}(\tilde{a} _{t})\}]\nonumber \\&=[\varkappa _{ij}(\tilde{a}_{t}),\ell _{ij}(\tilde{a}_{t})]\cup [\phi _{ij}^{\prime }(\tilde{a}_{t}),\eth _{ij}(\tilde{a}_{t})]\nonumber \\&=[\max \{ \varkappa _{ij}(\tilde{a}_{t}),\phi _{ij}^{\prime }(\tilde{a} _{t})\},\min \{ \ell _{ij}(\tilde{a}_{t}),\eth _{ij}(\tilde{a}_{t})\}]\nonumber \\&=[\phi _{ij}^{\prime }(\tilde{a}_{t}),\ell _{ij}(\tilde{a}_{t})]. \end{aligned}$$

15 $$\begin{aligned} ([ {\mathcal {L}} ]_{\hat{s}\times \breve{u}}\cup [{{\ss }}]_{\hat{s}\times \breve{u} })\cap ([ {\mathcal {L}} ]_{\hat{s}\times \breve{u}}\cup [{\text {\Thorn}}]_{\hat{s}\times \breve{u}})&=\left[ \begin{array}{c} \max \{ \varkappa _{ij}(\tilde{a}_{t}),\phi _{ij}^{\prime }(\tilde{a}_{t})\},\\ \min \{ \ell _{ij}(\tilde{a}_{t}),\phi _{ij}^{\prime \prime }(\tilde{a}_{t}) \end{array} \right] \cap \left[ \begin{array}{c} \max \{ \varkappa _{ij}(\tilde{a}_{t}),\Re _{ij}(\tilde{a}_{t})\},\\ \min \{ \ell _{ij}(\tilde{a}_{t}),\eth _{ij}(\tilde{a}_{t}) \end{array} \right] \nonumber \\&=[\phi _{ij}^{\prime }(\tilde{a}_{t}),\varkappa _{ij}(\tilde{a}_{t} )]\cap [\Re _{ij}(\tilde{a}_{t}),\ell _{ij}(\tilde{a}_{t})]\nonumber \\&=\left[ \begin{array}{c} \min \{ \phi _{ij}^{\prime }(\tilde{a}_{t}),\Re _{ij}(\tilde{a}_{t})\},\\ \max \{ \ell _{ij}(\tilde{a}_{t}),\ell _{ij}(\tilde{a}_{t})\} \end{array} \right] \nonumber \\&=[\phi _{ij}^{\prime }(\tilde{a}_{t}),\ell _{ij}(\tilde{a}_{t})]. \end{aligned}$$

From (14) and (15), we have

$$\begin{aligned}{}[{\mathcal {L}} ]_{\hat{s}\times \breve{u}}\cup ([{{\ss }}]_{\hat{s}\times \breve{u}} \cap [{\text {\Thorn}}]_{\hat{s}\times \breve{u}})=([ {\mathcal {L}} ]_{\hat{s}\times \breve{u}}\cup [{{\ss }}]_{\hat{s}\times \breve{u} })\cap ([ {\mathcal {L}} ]_{\hat{s}\times \breve{u}}\cup [{\text {\Thorn}}]_{\hat{s}\times \breve{u}}). \end{aligned}$$

*Case 2.* For all $[\varkappa _{ij}(\tilde{a}_{t}),\ell _{ij}(\tilde{a}_{t})]\in [{\mathcal {L}} ]_{\hat{s}\times \breve{u}}$, $[\phi _{ij}^{\prime }(\tilde{a}_{t}),\phi _{ij}^{\prime \prime }(\tilde{a}_{t})]\in [{{\ss }}]_{\hat{s} \times \breve{u}}$ and $[\Re _{ij}(\tilde{a}_{t}),\eth _{ij}(\tilde{a}_{t} )]\in [{\text {\Thorn}}]_{\hat{s}\times \breve{u}}$ such that $\varkappa _{ij}(\tilde{a}_{t})\le \Re _{ij}(\tilde{a}_{t})\le \phi _{ij}^{\prime } (\tilde{a}_{t})$ and $\ell _{ij}(\tilde{a}_{t})\le \eth _{ij}(\tilde{a}_{t} )\le \phi _{ij}^{\prime \prime }(\tilde{a}_{t})$ then

16 $$\begin{aligned}{}[{\mathcal {L}} ]_{\hat{s}\times \breve{u}}\cup ([{{\ss }}]_{\hat{s}\times \breve{u}} \cap [{\text {\Thorn}}]_{\hat{s}\times \breve{u}})&=[ {\mathcal {L}} ]_{\hat{s}\times \breve{u}}\cup [\min \{ \phi _{ij}^{\prime }(\tilde{a} _{t}),\Re _{ij}(\tilde{a}_{t})\}, \max \{ \phi _{ij}^{\prime \prime }(\tilde{a}_{t}),\eth _{ij}(\tilde{a} _{t})\}]\nonumber \\&=[\varkappa _{ij}(\tilde{a}_{t}),\ell _{ij}(\tilde{a}_{t})]\cup [\Re _{ij}(\tilde{a}_{t}),\phi _{ij}^{\prime \prime }(\tilde{a}_{t})]\nonumber \\&=[\max \{ \varkappa _{ij}(\tilde{a}_{t}),\Re _{ij}(\tilde{a}_{t})\},\min \{ \ell _{ij}(\tilde{a}_{t}),\eth _{ij}(\tilde{a}_{t})\}]\nonumber \\&=[\Re _{ij}(\tilde{a}_{t}),\ell _{ij}(\tilde{a}_{t})]. \end{aligned}$$

17 $$\begin{aligned} ([ {\mathcal {L}} ]_{\hat{s}\times \breve{u}}\cup [{{\ss }}]_{\hat{s}\times \breve{u} })\cap ([ {\mathcal {L}} ]_{\hat{s}\times \breve{u}}\cup [{\text {\Thorn}}]_{\hat{s}\times \breve{u}})&=\left[ \begin{array}{c} \max \{ \varkappa _{ij}(\tilde{a}_{t}),\phi _{ij}^{\prime }(\tilde{a}_{t})\},\\ \min \{ \ell _{ij}(\tilde{a}_{t}),\phi _{ij}^{\prime \prime }(\tilde{a}_{t}) \end{array} \right] \cap \left[ \begin{array}{c} \max \{ \varkappa _{ij}(\tilde{a}_{t}),\Re _{ij}(\tilde{a}_{t})\},\\ \min \{ \ell _{ij}(\tilde{a}_{t}),\eth _{ij}(\tilde{a}_{t}) \end{array} \right] \nonumber \\&=[\phi _{ij}^{\prime }(\tilde{a}_{t}),\ell _{ij}(\tilde{a}_{t})]\cap [\Re _{ij}(\tilde{a}_{t}),\ell _{ij}(\tilde{a}_{t})]\nonumber \\&=\left[ \begin{array}{c} \min \{ \phi _{ij}^{\prime }(\tilde{a}_{t}),\Re _{ij}(\tilde{a}_{t})\},\\ \max \{ \ell _{ij}(\tilde{a}_{t}),\ell _{ij}(\tilde{a}_{t})\} \end{array} \right] \nonumber \\&=[\Re _{ij}(\tilde{a}_{t}),\ell _{ij}(\tilde{a}_{t})]. \end{aligned}$$

From (16) and (17), we have

$$\begin{aligned}{}[{\mathcal {L}} ]_{\hat{s}\times \breve{u}}\cup ([{{\ss }}]_{\hat{s}\times \breve{u}} \cap [{\text {\Thorn}}]_{\hat{s}\times \breve{u}})=([ {\mathcal {L}} ]_{\hat{s}\times \breve{u}}\cup [{{\ss }}]_{\hat{s}\times \breve{u} })\cap ([ {\mathcal {L}} ]_{\hat{s}\times \breve{u}}\cup [{\text {\Thorn}}]_{\hat{s}\times \breve{u}}). \end{aligned}$$

**Case 3.** For all $[\varkappa _{ij}(\tilde{a}_{t}),\ell _{ij}(\tilde{a}_{t})]\in [{\mathcal {L}} ]_{\hat{s}\times \breve{u}}$, $[\phi _{ij}^{\prime }(\tilde{a}_{t}),\phi _{ij}^{\prime \prime }(\tilde{a}_{t})]\in [{{\ss }}]_{\hat{s} \times \breve{u}}$ and $[\Re _{ij}(\tilde{a}_{t}),\eth _{ij}(\tilde{a}_{t} )]\in [{\text {\Thorn}}]_{\hat{s}\times \breve{u}}$ such that $\Re _{ij} (\tilde{a}_{t})\le \phi _{ij}^{\prime }(\tilde{a}_{t})\le \varkappa _{ij} (\tilde{a}_{t})$and $\eth _{ij}(\tilde{a}_{t})\le \phi _{ij}^{\prime \prime }(\tilde{a}_{t})\le \ell _{ij}(\tilde{a}_{t})$ then

18 $$\begin{aligned}{}[{\mathcal {L}} ]_{\hat{s}\times \breve{u}}\cup ([{{\ss }}]_{\hat{s}\times \breve{u}} \cap [{\text {\Thorn}}]_{\hat{s}\times \breve{u}})&=[ {\mathcal {L}} ]_{\hat{s}\times \breve{u}}\cup [\min \{ \phi _{ij}^{\prime }(\tilde{a} _{t}),\Re _{ij}(\tilde{a}_{t})\}, \max \{ \phi _{ij}^{\prime \prime }(\tilde{a}_{t}),\eth _{ij}(\tilde{a} _{t})\}]\nonumber \\&=[\varkappa _{ij}(\tilde{a}_{t}),\ell _{ij}(\tilde{a}_{t})]\cup [\Re _{ij}(\tilde{a}_{t}),\phi _{ij}^{\prime \prime }(\tilde{a}_{t})]\nonumber \\&=[\max \{ \varkappa _{ij}(\tilde{a}_{t}),\Re _{ij}(\tilde{a}_{t})\},\min \{ \ell _{ij}(\tilde{a}_{t}),\phi _{ij}^{\prime \prime }(\tilde{a}_{t})\}]\nonumber \\&=[\varkappa _{ij}(\tilde{a}_{t}),\phi _{ij}^{\prime \prime }(\tilde{a}_{t})]. \end{aligned}$$

19 $$\begin{aligned} ([ {\mathcal {L}} ]_{\hat{s}\times \breve{u}}\cup [{{\ss }}]_{\hat{s}\times \breve{u} })\cap ([ {\mathcal {L}} ]_{\hat{s}\times \breve{u}}\cup [{\text {\Thorn}}]_{\hat{s}\times \breve{u}})&=\left[ \begin{array}{c} \max \{ \varkappa _{ij}(\tilde{a}_{t}),\phi _{ij}^{\prime }(\tilde{a}_{t})\},\\ \min \{ \ell _{ij}(\tilde{a}_{t}),\phi _{ij}^{\prime \prime }(\tilde{a}_{t}) \end{array} \right] \cap \left[ \begin{array}{c} \max \{ \varkappa _{ij}(\tilde{a}_{t}),\Re _{ij}(\tilde{a}_{t})\},\\ \min \{ \ell _{ij}(\tilde{a}_{t}),\eth _{ij}(\tilde{a}_{t}) \end{array} \right] \nonumber \\&=[\varkappa _{ij}(\tilde{a}_{t}),\phi _{ij}^{\prime \prime }(\tilde{a} _{t})]\cap [\varkappa _{ij}(\tilde{a}_{t}),\eth _{ij}(\tilde{a} _{t})]\nonumber \\&=\left[ \begin{array}{c} \min \{ \varkappa _{ij}(\tilde{a}_{t}),\varkappa _{ij}(\tilde{a}_{t})\},\\ \max \{ \phi _{ij}^{\prime \prime }(\tilde{a}_{t}),\eth _{ij}(\tilde{a}_{t})\} \end{array} \right] \nonumber \\&=[\varkappa _{ij}(\tilde{a}_{t}),\phi _{ij}^{\prime \prime }(\tilde{a}_{t})]. \end{aligned}$$

From (18) and (19), we have

$$\begin{aligned}{}[{\mathcal {L}} ]_{\hat{s}\times \breve{u}}\cup ([{{\ss }}]_{\hat{s}\times \breve{u}} \cap [{\text {\Thorn}}]_{\hat{s}\times \breve{u}})=([ {\mathcal {L}} ]_{\hat{s}\times \breve{u}}\cup [{{\ss }}]_{\hat{s}\times \breve{u} })\cap ([ {\mathcal {L}} ]_{\hat{s}\times \breve{u}}\cup [{\text {\Thorn}}]_{\hat{s}\times \breve{u}}). \end{aligned}$$

Thus, the distributive law of union over intersection holds in all cases.

(*ii*). By duality principle (*ii*) is also hold.

## Distance measures of complex Pythagorean fuzzy soft matrices

In this section, we will define a novel distance measure for CPFSMs. Distance measures of CPFSMs have far-reaching significance and applications in various fields, including decision-making, uncertainty analysis, and data analytics. These measures enable the assessment of similarity and dissimilarity between CPFSMs, which is crucial for evaluating alternative solutions, identifying patterns, and optimizing complex systems. In decision-making, distance measures of CPFSMs help select the most suitable option by quantifying the proximity between alternatives. In uncertainty analysis, they facilitate the evaluation of uncertainty levels and risk assessment. Additionally, distance measures of CPFSMs have applications in image processing, natural language processing, and recommender systems, which aid in image segmentation, text classification, and personalized recommendations. Furthermore, they are useful in social network analysis, clustering, and classification, enabling the identification of influential nodes, clusters, and patterns. Overall, distance measures of CPFSMs provide a powerful tool for analyzing and understanding complex data, leading to more informed decisions and improved outcomes.

### Definition 5.1

***A distance measure of CPFSMs is a function $\amalg :M_{\hat{s}\times \breve{u} }\times M_{\hat{s}\times \breve{u}}\rightarrow [0,1]$ with the properties: for any $[ {\mathcal {L}} ]_{\hat{s}\times \breve{u}}=[\varkappa _{ij}(q_{t}),\ell _{ij}(q_{t})], [{{\ss}}]_{\hat{s}\times \breve{u}}=[\phi _{ij}^{\prime }(q_{t}),\phi _{ij}^{\prime \prime }(q_{t})],$ and $[{\text {\Thorn}}]_{\hat{s}\times \breve{u}} =[\Re _{ij}(q_{t}),\eth _{ij}(q_{t})]\in M_{\hat{s}\times \breve{u}}$

*i*).: $0\le \amalg ([ {\mathcal {L}} ]_{\hat{s}\times \breve{u}},[{{\ss }}]_{\hat{s}\times \breve{u}})\le 1,$ $\amalg ([ {\mathcal {L}} ]_{\hat{s}\times \breve{u}},[{{\ss }}]_{\hat{s}\times \breve{u}})=0$ if and only if $[ {\mathcal {L}} ]_{\hat{s}\times \breve{u}}=[{{\ss }}]_{\hat{s}\times \breve{u}}.$
*ii*).: $$\amalg ([ {\mathcal {L}} ]_{\hat{s}\times \breve{u}},[{{\ss }}]_{\hat{s}\times \breve{u}})=\amalg ([{{\ss }}]_{\hat{s}\times \breve{u}},[ {\mathcal {L}} ]_{\hat{s}\times \breve{u}}).$$
*iii*).: $$\amalg ([ {\mathcal {L}} ]_{\hat{s}\times \breve{u}},[{\text {\Thorn}}]_{\hat{s}\times \breve{u}})\le \amalg ([ {\mathcal {L}} ]_{\hat{s}\times \breve{u}},[{{\ss }}]_{\hat{s}\times \breve{u}})+\amalg ([{{\ss }}]_{\hat{s}\times \breve{u}},[{\text {\Thorn}}]_{\hat{s}\times \breve{u}}).$$

We introduce the distance measure $\amalg$ as:

20 $$\begin{aligned} \amalg ([ {\mathcal {L}} ]_{\hat{s}\times \breve{u}},[{{\ss }}]_{\hat{s}\times \breve{u}})=\frac{1}{2\breve{u}}\underset{t,j=1}{\overset{\breve{u}}{\sum }}\left[ \begin{array}{c} |\underset{j}{\max }\varkappa _{ij}(\tilde{a}_{t})-\underset{j}{\max }\phi _{ij}^{\prime }(\tilde{a}_{t})|\\+|\underset{j}{\min }\ell _{ij}(\tilde{a}_{t})-\underset{j}{\min }\phi _{ij}^{\prime \prime }(\tilde{a}_{t})| \end{array} \right] . \end{aligned}$$

Note that the distance measure $\amalg$ plays a key role in the remainder of this paper.

### Example 5.1

Let

$$[ {\mathcal {L}} _{2\times 2}]= \begin{pmatrix} [0.5,0.8] &{} [0.4,0.6]\\ [0.1,0.7] &{} [0.3,0.9] \end{pmatrix} $$ and [sss $$_{2\times 2}]= \begin{pmatrix} [0.8,0.3] &{} [0.6,0.5]\\ [0.4,0.8] &{} [0.2,0.7] \end{pmatrix} $$ then

$$\begin{aligned} \amalg ([ {\mathcal {L}} ]_{\hat{s}\times \breve{u}},[{{\ss }}]_{\hat{s}\times \breve{u}})&=\frac{1}{2(2)}\left[ \left( |0.5-0.8|+|0.7-0.3|\right) +\left( |0.4-0.6|+|0.6-0.5|\right) \right] \\&=\frac{1}{4}\left[ 0.3+0.4+0.2+0.1\right] \\&=0.25. \end{aligned}$$

### Theorem 5.2

The function $\amalg$ defined by the equality (20) is a distance measure of CPFSMs on U.

### Proof

The condition $\amalg ([ {\mathcal {L}} ]_{\hat{s}\times \breve{u}},[{{\ss }}]_{\hat{s}\times \breve{u}})\ge 0$ obviously holds. Next, consider

$$\begin{aligned} \amalg ([ {\mathcal {L}} ]_{\hat{s}\times \breve{u}},[{{\ss }}]_{\hat{s}\times \breve{u}})&=\frac{1}{2\breve{u}}\underset{t,j=1}{\overset{\breve{u}}{\sum }}\left[ \begin{array}{c} |\underset{j}{\max }\varkappa _{ij}(\tilde{a}_{t})-\underset{j}{\max }\phi _{ij}^{\prime }(\tilde{a}_{t})|\\ +|\underset{j}{\min }\ell _{ij}(\tilde{a}_{t})-\underset{j}{\min }\phi _{ij}^{\prime \prime }(\tilde{a}_{t})| \end{array} \right] \\&=\frac{1}{2\breve{u}}\underset{t,j=1}{\overset{\breve{u}}{\sum }}\left[ 1+1\right] \\&=\frac{1}{2\breve{u}}(2)\\&=\frac{1}{\breve{u}}\le 1. \end{aligned}$$

Therefore $$\amalg ([ {\mathcal {L}} ]_{\hat{s}\times \breve{u}},[{{\ss }}]_{\hat{s}\times \breve{u}})\le 1.$$

Note that if $\breve{u}=1,$ then $\amalg ([ {\mathcal {L}} ]_{\hat{s}\times \breve{u}}, [{{\ss }}]_{\hat{s}\times \breve{u}})=1.$

$$\begin{aligned} \amalg ([ {\mathcal {L}} ]_{\hat{s}\times \breve{u}},[ {\mathcal {L}} ]_{\hat{s}\times \breve{u}})&=\frac{1}{2\breve{u}}\underset{t,j=1}{\overset{\breve{u}}{\sum }}\left[ \begin{array}{c} |\underset{j}{\max }\varkappa _{ij}(\tilde{a}_{t})-\underset{j}{\max } \varkappa _{ij}(\tilde{a}_{t})|\\ +|\underset{j}{\min }\ell _{ij}(\tilde{a}_{t})-\ell _{ij}(\tilde{a}_{t})| \end{array} \right] \\&=\frac{1}{2\breve{u}}\underset{t,j=1}{\overset{\breve{u}}{\sum }}\left[ 0+0\right] \\&=\frac{1}{2\breve{u}}(0)\\&=0. \end{aligned}$$

Condition (ii) is straightforward. To prove (iii), we have

$$\begin{aligned} \amalg ([ {\mathcal {L}} ]_{\hat{s}\times \breve{u}},[{\text {\Thorn}}]_{\hat{s}\times \breve{u}})&=\frac{1}{2\breve{u}}\underset{t,j=1}{\overset{\breve{u}}{\sum }}\left[ \begin{array}{c} |\underset{j}{\max }\varkappa _{ij}(\tilde{a}_{t})-\underset{j}{\max }\Re _{ij}(\tilde{a}_{t})|\\ +|\underset{j}{\min }\ell _{ij}(\tilde{a}_{t})-\underset{j}{\min }\eth _{ij} (\tilde{a}_{t})| \end{array} \right] \\&=\frac{1}{2\breve{u}}\underset{t,j=1}{\overset{\breve{u}}{\sum }}\left[ \begin{array}{c} |\underset{j}{\max }\varkappa _{ij}(\tilde{a}_{t})-\underset{j}{\max }\phi _{ij}^{\prime }(\tilde{a}_{t})\\ +\underset{j}{\max }\phi _{ij}^{\prime }(\tilde{a}_{t})-\underset{j}{\max }\Re _{ij}(\tilde{a}_{t})|\\ +|\underset{j}{\min }\ell _{ij}(\tilde{a}_{t})-\underset{j}{\min }\phi _{ij}^{\prime \prime }(\tilde{a}_{t})\\ +\underset{j}{\min }\phi _{ij}^{\prime \prime }(\tilde{a}_{t})-\underset{j}{\min }\eth _{ij}(\tilde{a}_{t})| \end{array} \right] \\&\le \frac{1}{2\breve{u}}\underset{t,j=1}{\overset{\breve{u}}{\sum }}\left[ \begin{array}{c} |\underset{j}{\max }\varkappa _{ij}(\tilde{a}_{t})-\underset{j}{\max }\phi _{ij}^{\prime }(\tilde{a}_{t})|\\ +|\underset{j}{\max }\phi _{ij}^{\prime }(\tilde{a}_{t})-\underset{j}{\max } \Re _{ij}(\tilde{a}_{t})|\\ +|\underset{j}{\min }\ell _{ij}(\tilde{a}_{t})-\underset{j}{\min }\phi _{ij}^{\prime \prime }(\tilde{a}_{t})|\\ +|\underset{j}{\min }\phi _{ij}^{\prime \prime }(\tilde{a}_{t})-\underset{j}{\min }\eth _{ij}(\tilde{a}_{t})| \end{array} \right] \\&=\frac{1}{2\breve{u}}\underset{t,j=1}{\overset{\breve{u}}{\sum }}\left[ \begin{array}{c} |\underset{j}{\max }\varkappa _{ij}(\tilde{a}_{t})-\underset{j}{\max }\phi _{ij}^{\prime }(\tilde{a}_{t})|\\ +|\underset{j}{\min }\ell _{ij}(\tilde{a}_{t})-\underset{j}{\min }\phi _{ij}^{\prime \prime }(\tilde{a}_{t})| \end{array} \right] +\frac{1}{2\breve{u}}\underset{t,j=1}{\overset{\breve{u}}{\sum }}\left[ \begin{array}{c} |\underset{j}{\max }\phi _{ij}^{\prime }(\tilde{a}_{t})-\underset{j}{\max } \Re _{ij}(\tilde{a}_{t})|\\ +|\underset{j}{\min }\phi _{ij}^{\prime \prime }(\tilde{a}_{t})-\underset{j}{\min }\eth _{ij}(\tilde{a}_{t})| \end{array} \right] \\&=\amalg ([ {\mathcal {L}} ]_{\hat{s}\times \breve{u}},[{{\ss }}]_{\hat{s}\times \breve{u}} )+\amalg ([{{\ss }}]_{\hat{s}\times \breve{u}},[{\text {\Thorn}}]_{\hat{s} \times \breve{u}}). \end{aligned}$$

Hence $$\amalg ([ {\mathcal {L}} ]_{\hat{s}\times \breve{u}},[{\text {\Thorn}}]_{\hat{s}\times \breve{u}})\le \amalg ([ {\mathcal {L}} ]_{\hat{s}\times \breve{u}},[{{\ss }}]_{\hat{s}\times \breve{u}})+\amalg ([{{\ss }}]_{\hat{s}\times \breve{u}},[{\text {\Thorn}}]_{\hat{s}\times \breve{u}}).$$$$\square $$

### Definition 5.2

A weighted distance measure of CPFSMs is a function $\amalg _{w}:M_{\hat{s}\times \breve{u}}\times M_{\hat{s}\times \breve{u}}\rightarrow [0,1]$ with the properties: for any $[ {\mathcal {L}} ]_{\hat{s}\times \breve{u}}=[\varkappa _{ij}(\tilde{a}_{t}),\ell _{ij}(\tilde{a}_{t})], [{{\ss }}]_{\hat{s}\times \breve{u}}=[\phi _{ij}^{\prime }(\tilde{a}_{t}),\phi _{ij}^{\prime \prime }(\tilde{a}_{t})],$ and ${{\text {\Thorn}}}_{\hat{s}\times \breve{u}}=[\Re _{ij}(\tilde{a}_{t}),\eth _{ij}(\tilde{a}_{t})]\in \hat{s}_{\hat{s}\times \breve{u}}$

*i*.: $0\le \amalg _{w}([ {\mathcal {L}} ]_{\hat{s}\times \breve{u}},[{{\ss }}]_{\hat{s}\times \breve{u}})\le 1,$ $\amalg _{w}([ {\mathcal {L}} ]_{\hat{s}\times \breve{u}},[{{\ss }}]_{\hat{s}\times \breve{u}})=0$ if and only if $[ {\mathcal {L}} ]_{\hat{s}\times \breve{u}}=[{{\ss }}]_{\hat{s}\times \breve{u}}.$
*ii*.: $$\amalg _{w}([ {\mathcal {L}} ]_{\hat{s}\times \breve{u}},[{{\ss }}]_{\hat{s}\times \breve{u}})=\amalg _{w}([{{\ss }}]_{\hat{s}\times \breve{u}},[ {\mathcal {L}} ]_{\hat{s}\times \breve{u}}).$$
*iii*.: $$\amalg _{w}([ {\mathcal {L}} ]_{\hat{s}\times \breve{u}},[{\text {\Thorn}}]_{\hat{s}\times \breve{u}})\le \amalg _{w}([ {\mathcal {L}} ]_{\hat{s}\times \breve{u}},[{{\ss }}]_{\hat{s}\times \breve{u}})+\amalg _{w} ([{{\ss }}]_{\hat{s}\times \breve{u}},[{\text {\Thorn}}]_{\hat{s}\times \breve{u}}).$$

We introduce the weighted distance measure $\amalg _{w}$ as:

21 $$\begin{aligned} \amalg _{w}([ {\mathcal {L}} ]_{\hat{s}\times \breve{u}},[{{\ss }}]_{\hat{s}\times \breve{u}})=\frac{1}{2\breve{u}}\underset{t,j=1}{\overset{\breve{u}}{\sum }}\left[ w_{t}\left( \begin{array}{c} |\underset{j}{\max }\varkappa _{ij}(\tilde{a}_{t})-\underset{j}{\max }\phi _{ij}^{\prime }(\tilde{a}_{t})|\\ +|\underset{j}{\min }\ell _{ij}(\tilde{a}_{t})-\underset{j}{\min }\phi _{ij}^{\prime \prime }(\tilde{a}_{t})| \end{array} \right) \right] \end{aligned}$$

where $w_{t}$ is a weighted vector corresponding to each parameter.

### Theorem 5.3

The function $\amalg$ defined by the equality (21) is a distance measure of CPFSMs on U.

### Proof

It is easy to prove. $\square$

## Applications of complex Pythagorean fuzzy soft matrix in decision-making

CPFSMs have many applications in DM problems due to their ability to handle uncertain, complex, and vague information. CPFSMs are applied in MCDM to evaluate alternatives based on multiple criteria. By incorporating degrees of membership and non-membership, CPFSMs can effectively model the uncertainty and ambiguity inherent in DM processes involving various criteria. CPFSMs provide a robust framework for solving, analyzing, and modeling complex DM problems, thereby enhancing the reliability and quality of decisions made in practical applications.

We discuss the subsequent definitions of a DM matrix, considering the notions of a CPFSM.

### Definition 6.1

Assume there are n evaluation objects (scheme set), $$\hbar =\{ \hbar _{1} ,\hbar _{2},...,\hbar _{n}\}$$ in a multiattribute DM problem and *m* evaluation attributes form an index set $$\wp =\{ \wp _{1},\wp _{2},...,\wp _{m}\}.$$ The attribute value of scheme $$\hbar _{i}$$ for index $$\wp _{j}$$ is $$[\varkappa _{ij}^{\prime },\varkappa _{ij}^{\prime \prime }]$$ $$(i=1,2,...,n$$ ;  $$j=1,2,...,m).$$ So the CPFS DM matrix of the scheme set $$\hbar $$ for the index set $$\wp $$ is

$$\begin{aligned} \chi = \begin{pmatrix} [\varkappa _{11}^{\prime },\varkappa _{11}^{\prime \prime }] &{} [\varkappa _{12}^{\prime },\varkappa _{12}^{\prime \prime }] &{}... &{} [\varkappa _{1n}^{\prime },\varkappa _{1n}^{\prime \prime }]\\ [\varkappa _{21}^{\prime },\varkappa _{21}^{\prime \prime }] &{} [\varkappa _{22}^{\prime },\varkappa _{22}^{\prime \prime }] &{}... &{} [\varkappa _{2n}^{\prime },\varkappa _{2n}^{\prime \prime }]\\ ... &{}... &{}... &{}...\\ [\varkappa _{m1}^{\prime },\varkappa _{m1}^{\prime \prime }] &{} [\varkappa _{m2}^{\prime },\varkappa _{m2}^{\prime \prime }] &{}... &{} [\varkappa _{mn}^{\prime },\varkappa _{mn}^{\prime \prime }] \end{pmatrix}. \end{aligned}$$

Here the terms $$\varkappa _{ij}^{\prime }$$ and $$\varkappa _{ij}^{\prime \prime }$$ $$(i=1,2,...,n$$ ;  $$j=1,2,...,m)$$ denote the truth grade (TG) and false grade (FG) of the CPFS decision matrix.

### Normalization process

In the normalization process, we normalize the TG and FG of the CPFS DM matrix as follows:

$$\begin{aligned} \kappa _{ij}^{\prime }=\frac{\varkappa _{ij}^{\prime }}{\overset{n}{\underset{i=1}{\sum }\varkappa _{ij}^{\prime }}},\text { }\kappa _{ij}^{\prime \prime } =\frac{\varkappa _{ij}^{\prime \prime }}{\overset{n}{\underset{i=1}{\sum }\varkappa _{ij}^{\prime \prime }}}. \end{aligned}$$

This normalization process transforms the CPFS DM matrix $\chi$ into CPFS DM matrix $\Gamma$:

$$\begin{aligned} \Gamma = \begin{pmatrix} [\kappa _{11}^{\prime },\kappa _{11}^{\prime \prime }] &{} [\kappa _{12} ^{\prime },\kappa _{12}^{\prime \prime }] &{}... &{} [\kappa _{1m}^{\prime },\kappa _{1m}^{\prime \prime }]\\ [\kappa _{21}^{\prime },\kappa _{21}^{\prime \prime }] &{} [\kappa _{22} ^{\prime },\kappa _{22}^{\prime \prime }] &{}... &{} [\kappa _{2m}^{\prime },\kappa _{2m}^{\prime \prime }]\\ ... &{}... &{}... &{}...\\ [\kappa _{n1}^{\prime },\kappa _{n1}^{\prime \prime }] &{} [\kappa _{n2} ^{\prime },\kappa _{n2}^{\prime \prime }] &{}... &{} [\kappa _{nm}^{\prime },\kappa _{nm}^{\prime \prime }] \end{pmatrix}. \end{aligned}$$

#### Definition 6.2

Assume that

$$\begin{aligned} \kappa _{i^{+}j}&=\max \left\{ \frac{\kappa _{i^{+}j}^{\prime }+\kappa _{i^{+}j}^{\prime \prime }}{2};\text { }for\text { fixed i}\right\} \\ j&=1,2,...,n\text { }; \end{aligned}$$

and thus

$$\begin{aligned}{}[\hbar _{i}^{+}]_{m\times n}= \begin{pmatrix} [\kappa _{1^{+}1}^{^{\prime }},\kappa _{1^{+}1}^{\prime \prime }]\\ [\kappa _{2^{+}1}^{\prime },\kappa _{2^{+}1}^{\prime \prime }]\\ .\\ .\\ .\\ [\kappa _{n^{+}1}^{\prime },\kappa _{n^{+}1}^{\prime \prime }] \end{pmatrix}, \end{aligned}$$

is called positive CPFS DM matrix for scheme $\hbar _{i}$.

#### Definition 6.3

Assume that

$$\begin{aligned} \kappa _{i^{-}j}&=\min \left\{ \frac{\kappa _{i^{-}j}^{\prime }+\kappa _{i^{-}j}^{\prime \prime }}{2};\text { }for\text { fixed i}\right\} \\ j&=1,2,...,n. \end{aligned}$$

and thus

$$\begin{aligned}{}[\hbar _{i}^{-}]_{m\times n}= \begin{pmatrix} [\kappa _{1^{+}1}^{\prime },\kappa _{1^{+}1}^{\prime \prime }]\\ [\kappa _{2^{+}1}^{\prime },\kappa _{2^{+}1}^{\prime \prime }]\\ .\\ .\\ .\\ [\kappa _{n^{+}1}^{\prime },\kappa _{n^{+}1}^{\prime \prime }] \end{pmatrix}, \end{aligned}$$

is called negative CPFS DM matrix for scheme $\hbar _{i}$.

#### Graphical representation of algorithm

**Figure 1:**
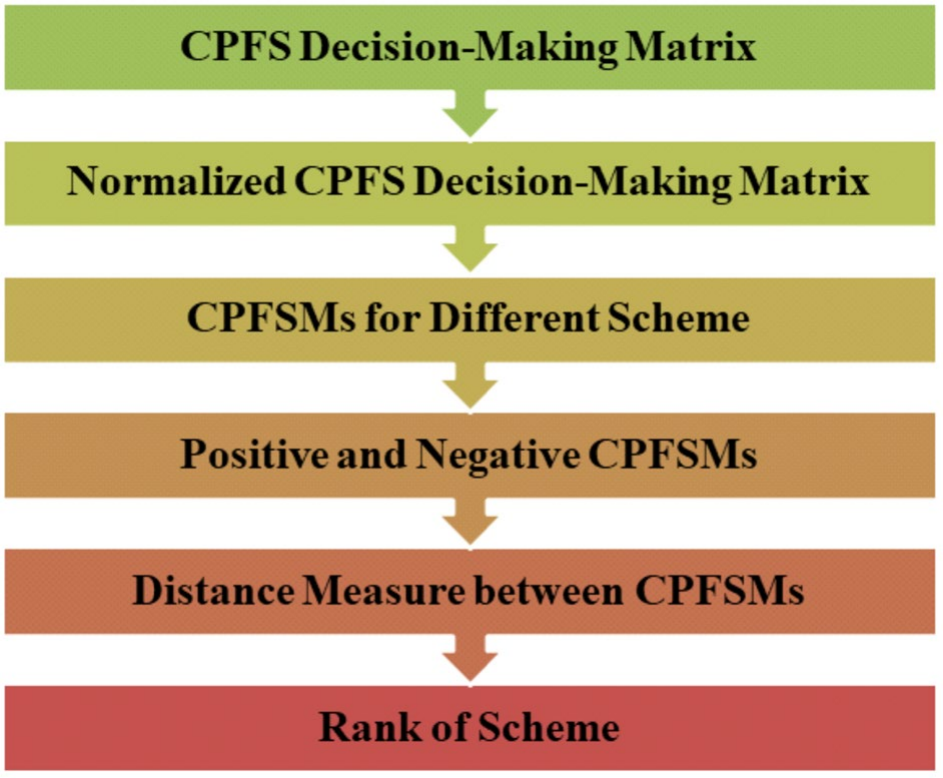
Graphical absract of algorithm.

 The graphical representation of an algorithm is given below in Fig. 1:

### Algorithm

Below are the steps outlining our algorithm for addressing a standard DM problem utilizing the CPFSM alongside positive and negative CPFS DM matrices.

*Step 1.*

Construct the CPFS DM matrix $\chi$ corresponding to CPFS sets.

*Step 2.*

Normalize the CPFS DM matrices $\chi _{i}$ into CPFS DM matrix $\Gamma _{i}.$

*Step 3.*

Compute the CPFSMs of each scheme $\hbar _{i}$.

*Step 4.*

Compute each scheme’s positive and negative CPFSMs $\hbar _{i}$.

*Step 5.*

Find the distance measures between positive and negative CPFS DM matrices.

*Step 6.*

Rank the scheme based on distance measures of CPFSMs.

The scheme will be better if the distance measures between positive and The scheme’s adverse CPFS decision-making matrices are more significant than the other distances.

### A case analysis

Suppose an iPhone mobile company invests in three enterprises (schemes). Let $\hbar =\{ \hbar _{1}=social$ *benefit* $index,\hbar _{2}=environment$ $pollution,\hbar _{3}=economic$ *benefit* $index\}.$ The expert team conducts investigations and participates in evaluating these three enterprises from 2018 to 2020. Let $\wp =\{ \wp _{1},\wp _{2},\wp _{3}\}$ be an index set. The expert team presents the evaluation results for each index as CPFS sets.

Now, for 2018, we have

$$[\chi (\hbar _{1})]=\{(\wp _{1},$$ $$(0.2e^{i\frac{\pi }{2}},0.5e^{i\pi })),(\wp _{2},(0.1e^{i\frac{3\pi }{2}},0.8e^{i2\pi })),(\wp _{3},(0.6e^{i\frac{\pi }{2} },0.8e^{i\pi }))\},$$

$$[\chi (\hbar _{2})]=\{(\wp _{1},$$ $$(0.5e^{i\frac{3\pi }{2}},0.7e^{i\pi } )),(\wp _{2},(0.4e^{i2\pi },0.6e^{i\frac{\pi }{2}})),(\wp _{3},(0.8e^{i\frac{\pi }{4}},0.3e^{i\frac{\pi }{2}}))\},$$

$$[\chi (\hbar _{3})]=\{(\wp _{1},$$ $$(0.4e^{i\pi },0.6e^{i2\pi })),(\wp _{2},(0.2e^{i\pi },0.8e^{i\frac{3\pi }{2}})),(\wp _{3},(0.5e^{i\frac{\pi }{2} },0.3e^{i\frac{\pi }{4}}))\}.$$

For 2019, we have

$$[\chi (\hbar _{1})]=\{(\wp _{1},$$ $$(1e^{i\frac{\pi }{2}},0e^{i\pi })),(\wp _{2},(0.8e^{i\frac{3\pi }{2}},0.3e^{i2\pi })),(\wp _{3},(0.7e^{i\frac{\pi }{2} },0.1e^{i\pi }))\},$$

$$[\chi (\hbar _{2})]=\{(\wp _{1},$$ $$(0.2e^{i\frac{3\pi }{2}},0.7e^{i\pi } )),(\wp _{2},(0.9e^{i2\pi },0.2e^{i\frac{\pi }{2}})),(\wp _{3},(0.7e^{i\frac{\pi }{4}},0.3e^{i\frac{\pi }{2}}))\},$$

$$[\chi (\hbar _{3})]=\{(\wp _{1},$$ $$(0.8e^{i\pi },0.6e^{i2\pi })),(\wp _{2},(0.5e^{i\pi },0.6e^{i\frac{3\pi }{2}})),(\wp _{3},(1e^{i\frac{\pi }{2} },0e^{i\frac{\pi }{4}}))\}.$$

For 2020, we have

$$[\chi (\hbar _{1})]=\{(\wp _{1},$$ $$(0.2e^{i\frac{\pi }{2}},0.7e^{i\pi })),(\wp _{2},(0.4e^{i\frac{3\pi }{2}},0.7e^{i2\pi })),(\wp _{3},(0.8e^{i\frac{\pi }{2} },0.3e^{i\pi }))\},$$

$$[\chi (\hbar _{2})]=\{(\wp _{1},$$ $$(0.5e^{i\frac{3\pi }{2}},0.8e^{i\pi } )),(\wp _{2},(1e^{i2\pi },0e^{i\frac{\pi }{2}})),(\wp _{3},(0.2e^{i\frac{\pi }{4} },0.9e^{i\frac{\pi }{2}}))\},$$

$$[\chi (\hbar _{3})]=\{(\wp _{1},$$ $$(0.4e^{i\pi },0.9e^{i2\pi })),(\wp _{2},(0.2e^{i\pi },0.8e^{i\frac{3\pi }{2}})),(\wp _{3},(0.5e^{i\frac{\pi }{2} },0e^{i\frac{\pi }{4}}))\}.$$

*Step 1.*

The CPFS DM matrix $\chi$ during the three years is given below:

$$\begin{aligned} \chi _{1}&= \begin{pmatrix} [0.2,0.5] &{} [0.5,0.7] &{} [0.4,0.6]\\ [0.1,0.8] &{} [0.4,0.6] &{} [0.2,0.8]\\ [0.6,0.8] &{} [0.8,0.3] &{} [0.5,0.3] \end{pmatrix} ,\\ \chi _{2}&= \begin{pmatrix} [1,0] &{} [0.2,0.7] &{} [0.8,0.6]\\ [0.8,0.3] &{} [0.9,0.2] &{} [0.5,0.6]\\ [0.7,0.1] &{} [0.7,0.3] &{} [1,0] \end{pmatrix} ,\\ \chi _{3}&= \begin{pmatrix} [0.2,0.7] &{} [0.5,0.8] &{} [0.4,0.9]\\ [0.4,0.7] &{} [1,0] &{} [0.2,0.8]\\ [0.8,0.3] &{} [0.2,0.9] &{} [0.5,0] \end{pmatrix} . \end{aligned}$$

*Step 2.*

Normalize the CPFS DM matrices $\chi _{i}$ into CPFS DM matrix $\Gamma _{i}.$

$$\begin{aligned} \Gamma _{1}&= \begin{pmatrix} [0.22,0.24] &{} [0.29,0.44] &{} [0.36,0.35]\\ [0.11,0.38] &{} [0.24,0.38] &{} [0.18,0.47]\\ [0.67,0.38] &{} [0.47,0.19] &{} [0.45,0.18] \end{pmatrix} , \end{aligned}$$

$$\begin{aligned} \Gamma _{2}&= \begin{pmatrix} [0.4,0] &{} [0.11,0.58] &{} [0.35,0.5]\\ [0.32,0.75] &{} [0.5,0.17] &{} [0.22,0.5]\\ [0.28,0.25] &{} [0.39,0.25] &{} [0.43,0] \end{pmatrix} ,\\ \Gamma _{3}&= \begin{pmatrix} [0.14,0.41] &{} [0.29,0.47] &{} [0.36,0.53]\\ [0.29,0.0.41] &{} [0.59,0] &{} [0.18,0.47]\\ [0.57,0.18] &{} [0.12,0.53] &{} [0.45,0] \end{pmatrix} . \end{aligned}$$

*Step 3.*

The CPFSMs of the scheme $\hbar _{1},$ $\hbar _{2},$ and $\hbar _{3}$ are:

$$\begin{aligned}{}[\hbar _{1}]_{3\times 3}&= \begin{pmatrix} [0.22,0.24] &{} [0.4,0] &{} [0.14,0.41]\\ [0.11,0.38] &{} [0.32,0.75] &{} [0.29,0.0.41]\\ [0.67,0.38] &{} [0.28,0.25] &{} [0.57,0.18] \end{pmatrix} ,\\ [\hbar _{2}]_{3\times 3}&= \begin{pmatrix} [0.29,0.44] &{} [0.11,0.58] &{} [0.29,0.47]\\ [0.24,0.38] &{} [0.5,0.17] &{} [0.59,0]\\ [0.47,0.19] &{} [0.39,0.25] &{} [0.12,0.53] \end{pmatrix} ,\\ [\hbar _{3}]_{3\times 3}&= \begin{pmatrix} [0.36,0.35] &{} [0.35,0.5] &{} [0.36,0.53]\\ [0.18,0.47] &{} [0.22,0.5] &{} [0.18,0.47]\\ [0.45,0.18] &{} [0.43,0] &{} [0.45,0] \end{pmatrix} . \end{aligned}$$

*Step 4.*

Following the definitions 6.2 and 6.3, we compute the positive and negative CPFS DM matrices of scheme $\hbar _{1},$ $\hbar _{2},$ and $\hbar _{3}$ i.e.,

$$\begin{aligned}{}[\hbar _{1}^{+}]_{3\times 3}&= \begin{pmatrix} [0.14,0.41]\\ [0.32,0.75]\\ [0.67,0.38] \end{pmatrix} ,[\hbar _{2}^{+}]_{3\times 3}= \begin{pmatrix} [0.29,0.47]\\ [0.5,0.17]\\ [0.47,0.19] \end{pmatrix} , [\hbar _{3}^{+}]_{3\times 3} = \begin{pmatrix} [0.36,0.53]\\ [0.22,0.5]\\ [0.45,0.18] \end{pmatrix} . \end{aligned}$$

$$\begin{aligned}{}[\hbar _{1}^{-}]_{3\times 3}&= \begin{pmatrix} [0.4,0]\\ [0.11,0.38]\\ [0.28,0.25] \end{pmatrix} ,[\hbar _{2}^{-}]_{3\times 3}= \begin{pmatrix} [0.11,0.58]\\ [0.59,0]\\ [0.39,0.25] \end{pmatrix} , [\hbar _{3}^{-}]_{3\times 3} = \begin{pmatrix} [0.36,0.35]\\ [0.18,0.47]\\ [0.43,0] \end{pmatrix} . \end{aligned}$$

*Step 5.*

The distance measures between positive and negative CPFS DM matrices of the schemes $\hbar _{1},$ $\hbar _{2},$ and $\hbar _{3}$ are:

$$\begin{aligned} \amalg ([\hbar _{1}^{+}]_{3\times 3},[\hbar _{1}^{-}]_{3\times 3})&=0.325\\ \amalg ([\hbar _{2}^{+}]_{3\times 3},[\hbar _{2}^{-}]_{3\times 3})&=0.13\\ \amalg ([\hbar _{3}^{+}]_{3\times 3},[\hbar _{3}^{-}]_{3\times 3})&=0.10. \end{aligned}$$

*Step 6.*

Since $\amalg ([\hbar _{1}^{+}]_{3\times 3},[\hbar _{1}^{-}]_{3\times 3} )>\amalg ([\hbar _{2}^{+}]_{3\times 3},[\hbar _{2}^{-}]_{3\times 3})>\amalg ([\hbar _{3}^{+}]_{3\times 3},[\hbar _{3}^{-}]_{3\times 3}).$ Therefore, we conclude that the scheme $\hbar _{1}=social$ *benefit* *index* is better choice for investment.

## Comparison analysis

Here, we have examined the comparison of the proposed CPFSMs with the intuitionistic fuzzy soft matrices (IFSMs)^27^ and CFSMs^20^. With the help of some restrictions on the proposed structure of CPFSSs and CPFSMs, it is proposed that it can be reduced to the environments of PFSMs and CFSMs. The comparison is demonstrated in Remarks 7.1 and 7.2. The numerical data in the environment of CFSMs is also considered. Moreover, we discussed the advantages of the proposed CPFSMs.

### Remark 7.1

^27^ The proposed distance measure of CPFSMs reduce to the the environment of IFSSs if we consider the imaginary part as zero in truth grade and false grade and take $0\le \varkappa _{ij}(\tilde{a}_{t})+\ell _{ij}(\tilde{a}_{t})\le 1$ and $0\le \phi _{ij}^{\prime }(\tilde{a}_{t} )+\phi _{ij}^{\prime \prime }(\tilde{a}_{t})\le 1$ then

22 $$\begin{aligned} \amalg ([ {\mathcal {L}} ]_{\hat{s}\times \breve{u}},[{{\ss }}]_{\hat{s}\times \breve{u}})=\frac{1}{2n}\underset{t,j=1}{\overset{\breve{u}}{\sum }}\left[ \begin{array}{c} |\underset{j}{\max }\varkappa _{ij}(\tilde{a}_{t})-\underset{j}{\max }\phi _{ij}^{\prime }(\tilde{a}_{t})|\\ +|\underset{j}{\min }\ell _{ij}(\tilde{a}_{t})-\underset{j}{\min }\phi _{ij}^{\prime \prime }(\tilde{a}_{t})| \end{array} \right] . \end{aligned}$$

Equation (22) represents the distance measure in the environment of IFSSs.

### Remark 7.2

^20^ The distance measure of CPFSMs reduces to the distance the measure of CFSMs if we considered the false grade of CPFSSs as zero, then we have

23 $$\begin{aligned} \amalg ([ {\mathcal {L}} ]_{m\times n},[{{\ss }}]_{m\times n})=\frac{1}{n}\underset{t,j=1}{\overset{n}{\sum }}\left[ |\underset{j}{\max }\varkappa _{ij}(\tilde{a} _{t})-\underset{j}{\max }\phi _{ij}^{\prime }(\tilde{a}_{t})|\right] , \end{aligned}$$

where $$\varkappa _{ij}(\tilde{a}_{t}),\phi _{ij}^{\prime }(\tilde{a}_{t} )\in [0,1].$$

The distance measure mentioned in Remark 1 can be employed in DM problems. Now, let’s explore the DM problem within the context of IFSMs.

The intuitionistic fuzzy soft (IFS) information regarding the evaluation of the index is provided below.

Now, for 2018, we have

$$[\chi (\hbar _{1})]=\{(\wp _{1},$$ $$(0.2,0.5)),(\wp _{2},(0.1,0.8)),(\wp _{3},(0.3,0.5))\},$$

$$[\chi (\hbar _{2})]=\{(\wp _{1},$$ $$(0.5,0.4)),(\wp _{2},(0.4,0.6)),(\wp _{3},(0.8,0.1))\},$$

$$[\chi (\hbar _{3})]=\{(\wp _{1},$$ $$(0.4,0.6)),(\wp _{2},(0.2,0.8)),(\wp _{3},(0.5,0.3))\}.$$

For 2019, we have

$$[\chi (\hbar _{1})]=\{(\wp _{1},$$ $$(1,0)),(\wp _{2},(0.7,0.3)),(\wp _{3},(0.7,0.1))\},$$

$$[\chi (\hbar _{2})]=\{(\wp _{1},$$ $$(0.2,0.7)),(\wp _{2},(0.6,0.2)),(\wp _{3},(0.7,0.3))\},$$

$$[\chi (\hbar _{3})]=\{(\wp _{1},$$ $$(0.8,0.1)),(\wp _{2},(0.3,0.6)),(\wp _{3},(1,0))\}.$$

For 2020, we have

$$[\chi (\hbar _{1})]=\{(\wp _{1},$$ $$(0.2,0.7)),(\wp _{2},(0.1,0.7)),(\wp _{3},(0.6,0.3))\},$$

$$[\chi (\hbar _{2})]=\{(\wp _{1},$$ $$(0.5,0.2)),(\wp _{2},(1,0)),(\wp _{3},(0.2,0.8))\},$$

$$[\chi (\hbar _{3})]=\{(\wp _{1},$$ $$(0.4,0.6)),(\wp _{2},(0.2,0.8)),(\wp _{3},(0.5,0))\}.$$

The IFS DM matrix $\chi$ during the three years are given below:

$$\begin{aligned} \chi _{1}&= \begin{pmatrix} [0.2,0.5] &{} [0.5,0.4] &{} [0.4,0.6]\\ [0.1,0.8] &{} [0.4,0.6] &{} [0.2,0.8]\\ [0.3,0.5] &{} [0.8,0.1] &{} [0.5,0.3] \end{pmatrix} ,\\ \chi _{2}&= \begin{pmatrix} [1,0] &{} [0.2,0.7] &{} [0.8,0.1]\\ [0.7,0.3] &{} [0.6,0.2] &{} [0.3,0.6]\\ [0.7,0.1] &{} [0.7,0.3] &{} [1,0] \end{pmatrix} ,\\ \chi _{3}&= \begin{pmatrix} [0.2,0.7] &{} [0.5,0.2] &{} [0.4,0.6]\\ [0.1,0.7] &{} [1,0] &{} [0.2,0.8]\\ [0.6,0.3] &{} [0.2,0.8] &{} [0.5,0] \end{pmatrix} . \end{aligned}$$

Normalize the IFS DM matrices $\chi _{i}$ into IFS DM matrix $\Gamma _{i}.$

$$\begin{aligned} \Gamma _{1}&= \begin{pmatrix} [0.33,0.28] &{} [0.29,0.36] &{} [0.36,0.35]\\ [0.17,0.44] &{} [0.24,0.55] &{} [0.18,0.47]\\ [0.5,0.28] &{} [0.47,0.09] &{} [0.45,0.18] \end{pmatrix} ,\\ \Gamma _{2}&= \begin{pmatrix} [0.42,0] &{} [0.13,0.58] &{} [0.38,0.14]\\ [0.29,0.75] &{} [0.4,0.17] &{} [0.14,0.86]\\ [0.29,0.25] &{} [0.47,0.25] &{} [0.05,0] \end{pmatrix} ,\\ \Gamma _{3}&= \begin{pmatrix} [0.22,0.41] &{} [0.29,0.2] &{} [0.36,0.43]\\ [0.11,0.0.41] &{} [0.59,0] &{} [0.18,0.57]\\ [0.67,0.18] &{} [0.12,0.8] &{} [0.45,0] \end{pmatrix} . \end{aligned}$$

The IFSMs of the scheme $\hbar _{1},$ $\hbar _{2},$ and $\hbar _{3}$ are:

$$\begin{aligned}{}[\hbar _{1}]_{3\times 3}&= \begin{pmatrix} [0.33,0.28] &{} [0.42,0] &{} [0.22,0.41]\\ [0.17,0.44] &{} [0.29,0.75] &{} [0.11,0.0.41]\\ [0.5,0.28] &{} [0.29,0.25] &{} [0.67,0.18] \end{pmatrix} ,\\ [\hbar _{2}]_{3\times 3}&= \begin{pmatrix} [0.29,0.36] &{} [0.13,0.58] &{} [0.29,0.2]\\ [0.24,0.55] &{} [0.4,0.17] &{} [0.59,0]\\ [0.47,0.09] &{} [0.47,0.25] &{} [0.12,0.8] \end{pmatrix} ,\\ [\hbar _{3}]_{3\times 3}&= \begin{pmatrix} [0.36,0.35] &{} [0.38,0.14] &{} [0.36,0.43]\\ [0.18,0.47] &{} [0.14,0.86] &{} [0.18,0.57]\\ [0.45,0.18] &{} [0.05,0] &{} [0.45,0] \end{pmatrix} . \end{aligned}$$

Following the definitions 6.2 and 6.3, we compute the positive and negative IFS DM matrices of scheme $\hbar _{1},$ $\hbar _{2},$ and $\hbar _{3}$ i.e.,

$$\begin{aligned}{}[\hbar _{1}^{+}]_{3\times 3}&= \begin{pmatrix} [0.22,0.41]\\ [0.29,0.75]\\ [0.67,0.18] \end{pmatrix} ,[\hbar _{2}^{+}]_{3\times 3}= \begin{pmatrix} [0.13,0.58]\\ [0.24,0.55]\\ [0.12,0.8] \end{pmatrix} ,\\ [\hbar _{3}^{+}]_{3\times 3}&= \begin{pmatrix} [0.36,0.43]\\ [0.14,0.86]\\ [0.45,0.18] \end{pmatrix} . \\ [\hbar _{1}^{-}]_{3\times 3}&= \begin{pmatrix} [0.42,0]\\ [0.11,0.0.41]\\ [0.29,0.25] \end{pmatrix} ,[\hbar _{2}^{-}]_{3\times 3}= \begin{pmatrix} [0.29,0.2]\\ [0.4,0.17]\\ [0.47,0.09] \end{pmatrix} ,\\ [\hbar _{3}^{-}]_{3\times 3}&= \begin{pmatrix} [0.38,0.14]\\ [0.18,0.47]\\ [0.05,0] \end{pmatrix} . \end{aligned}$$

The distance measures between positive and negative IFS DM matrices of the schemes $\hbar _{1},$ $\hbar _{2},$ and $\hbar _{3}$ are:

$$\begin{aligned} \amalg ([\hbar _{1}^{+}]_{3\times 3},[\hbar _{1}^{-}]_{3\times 3})&=0.28,\\ \amalg ([\hbar _{2}^{+}]_{3\times 3},[\hbar _{2}^{-}]_{3\times 3})&=0.18,\\ \amalg ([\hbar _{3}^{+}]_{3\times 3},[\hbar _{3}^{-}]_{3\times 3})&=0.12. \end{aligned}$$

Since $\amalg ([\hbar _{1}^{+}]_{3\times 3},[\hbar _{1}^{-}]_{3\times 3} )>\amalg ([\hbar _{2}^{+}]_{3\times 3},[\hbar _{2}^{-}]_{3\times 3})>\amalg ([\hbar _{3}^{+}]_{3\times 3},[\hbar _{3}^{-}]_{3\times 3}).$ Therefore, we conclude that the scheme $\hbar _{1}=social$ *benefit* *index* is better choice for investment.

The distance measure mentioned in Remark 2 can be employed in DM problems. Now, let’s explore the DM problem within the context of CFSMs.

The complex fuzzy soft information regarding the evaluation of the index is provided below.

$$\begin{aligned}{}&\begin{array}{c|ccc} 2018 &{} \hbar _{1} &{} \hbar _{2} &{} \hbar _{3}\\ \hline \wp _{1} &{} 0.2e^{i\frac{\pi }{2}} &{} 0.5e^{i\frac{3\pi }{2}} &{} 0.4e^{i\pi } \\ \wp _{2} &{} 0.1e^{i\frac{3\pi }{2}} &{} 0.4e^{i2\pi } &{} 0.2e^{i\pi }\\ \wp _{3} &{} 0.6e^{i\frac{\pi }{2}} &{} 0.8e^{i\frac{\pi }{4}} &{} 0.5e^{i\frac{\pi }{2}} \end{array} \ , \begin{array}{c|ccc} 2019 &{} \hbar _{1} &{} \hbar _{2} &{} \hbar _{3}\\ \hline \wp _{1} &{} 1e^{i\frac{\pi }{2}} &{} 0.2e^{i\frac{3\pi }{2}} &{} 0.8e^{i\pi }\\ \wp _{2} &{} 0.8e^{i\frac{3\pi }{2}} &{} 0.9e^{i2\pi } &{} 0.5e^{i\pi }\\ \wp _{3} &{} 0.7e^{i\frac{\pi }{2}} &{} 0.7e^{i\frac{\pi }{4}} &{} 1e^{i\frac{\pi }{2}} \end{array} \ ,\\&\begin{array}{c|ccc} 2020 &{} \hbar _{1} &{} \hbar _{2} &{} \hbar _{3}\\ \hline \wp _{1} &{} 0.2e^{i\frac{\pi }{2}} &{} 0.5e^{i\frac{3\pi }{2}} &{} 0.4e^{i\pi } \\ \wp _{2} &{} 0.4e^{i\frac{3\pi }{2}} &{} 1e^{i2\pi } &{} 0.2e^{i\pi }\\ \wp _{3} &{} 0.8e^{i\frac{\pi }{2}} &{} 0.2e^{i\frac{\pi }{4}} &{} 0.5e^{i\frac{\pi }{2}} \end{array} \ . \end{aligned}$$

The CFS DM matrix $\chi$ during the three years is given below:

$$\begin{aligned} \chi _{1}&= \begin{pmatrix} 0.2 &{} 0.5 &{} 0.4\\ 0.1 &{} 0.4 &{} 0.2\\ 0.6 &{} 0.8 &{} 0.5 \end{pmatrix} ,\text { }\chi _{2}= \begin{pmatrix} 1 &{} 0.2 &{} 0.8\\ 0.8 &{} 0.9 &{} 0.5\\ 0.7 &{} 0.7 &{} 1 \end{pmatrix} ,\\ \chi _{3}&= \begin{pmatrix} 0.2 &{} 0.5 &{} 0.4\\ 0.4 &{} 1 &{} 0.2\\ 0.8 &{} 0.2 &{} 0.5 \end{pmatrix} . \end{aligned}$$

Normalize the CFS DM matrices $\chi _{i}$ into CFS DM matrix $\Gamma _{i}.$

$$\begin{aligned} \Gamma _{1}&= \begin{pmatrix} 0.22 &{} 0.29 &{} 0.36\\ 0.11 &{} 0.24 &{} 0.18\\ 0.67 &{} 0.47 &{} 0.45 \end{pmatrix} ,\text { }\Gamma _{2}= \begin{pmatrix} 0.4 &{} 0.11 &{} 0.35\\ 0.32 &{} 0.5 &{} 0.22\\ 0.28 &{} 0.39 &{} 0.43 \end{pmatrix} ,\\ \Gamma _{3}&= \begin{pmatrix} 0.14 &{} 0.29 &{} 0.36\\ 0.29 &{} 0.59 &{} 0.18\\ 0.57 &{} 0.12 &{} 0.45 \end{pmatrix} . \end{aligned}$$

The positive and negative CPFS DM matrices of scheme $\hbar _{1},$ $\hbar _{2},$ and $\hbar _{3}$ i.e.,

$$\begin{aligned}{} & {} [\hbar _{1}^{+}]_{3\times 3}= \begin{pmatrix} 0.36\\ 0.24\\ 0.67 \end{pmatrix},[\hbar _{2}^{+}]_{3\times 3}= \begin{pmatrix} 0.4\\ 0.5\\ 0.43 \end{pmatrix},[\hbar _{3}^{+}]_{3\times 3}= \begin{pmatrix} 0.36\\ 0.59\\ 0.57 \end{pmatrix}. \\{} & {} [\hbar _{1}^{-}]_{3\times 3}= \begin{pmatrix} 0.22\\ 0.11\\ 0.45 \end{pmatrix},[\hbar _{2}^{-}]_{3\times 3}= \begin{pmatrix} 0.11\\ 0.22\\ 0.28 \end{pmatrix},\text { }[\hbar _{3}^{-}]_{3\times 3}= \begin{pmatrix} 0.14\\ 0.18\\ 0.12 \end{pmatrix}. \end{aligned}$$

The distance measures between positive and negative CFS decision-making matrices of the schemes $\hbar _{1},$ $\hbar _{2},$ and $\hbar _{3}$ are:

$$\begin{aligned} \amalg ([\hbar _{1}^{+}]_{3\times 3},[\hbar _{1}^{-}]_{3\times 3})&=0.22\\ \amalg ([\hbar _{2}^{+}]_{3\times 3},[\hbar _{2}^{-}]_{3\times 3})&=0.22\\ \amalg ([\hbar _{3}^{+}]_{3\times 3},[\hbar _{3}^{-}]_{3\times 3})&=0.41 \end{aligned}$$

Therefore, we conclude that the scheme $\hbar _{3}$ is a better choice for investment.

problems, particularly in dynamic and uncertain environments.

## Conclusion

Complex fuzzy soft matrices and CPFSMs are both mathematical tools used for handling uncertainty and complexity in decision-making problems. But CPFSMs handle membership and non-membership functions, which can capture more complex and nuanced uncertainty. The novel notions of CPFSMs were defined in this paper. Basic CPFSM laws and attributes were explored. The set-theoretic operations, particular examples, and main results on CPFSMs were considered. A few novel distance metrics between two CPFSMs were presented by us. In the context of CPFSSs and CPFSMs, we developed a CPFS DM technique. We talked about how CPFSMs have been used to decision-making difficulties, with encouraging findings that have produced more precise and knowledgeable decision-making outcomes. Additionally, the comparison analysis of CFSMs and CPFSMs was presented. CPFSMs offer more advance and flexible tools for handling complex uncertainty, making them a better choice for intricate decision-making problems, while CFSMs remain suitable for relatively simpler uncertainty scenarios.

In the future, the researchers intend to expand upon the discussed work to encompass interval-valued CPFSMs, complex neutrosophic soft matrices, and other related areas, aiming to enhance the quality of their research endeavors.

## Data Availability

The datasets used and/or analyzed during the current study available from the corresponding author on reason-able request.
